# Marine Bioactive Peptides—An Overview of Generation, Structure and Application with a Focus on Food Sources

**DOI:** 10.3390/md18080424

**Published:** 2020-08-13

**Authors:** Milica Pavlicevic, Elena Maestri, Marta Marmiroli

**Affiliations:** 1 Institute for Food Technology and Biochemistry, Faculty of Agriculture, University of Belgrade, 11070 Belgrade, Serbia; mpavlicevic@agrif.bg.ac.rs; 2 Department of Chemistry, Life Sciences and Environmental Sustainability, and SITEIA.PARMA, University of Parma, 42123 Parma, Italy; marta.marmiroli@unipr.it; 3 Consorzio Italbiotec, Via Fantoli 16/15, 20138 Milan, Italy

**Keywords:** bioactive peptides, marine, secondary structure, proline, mechanism of activity, marine waste

## Abstract

The biggest obstacles in the application of marine peptides are two-fold, as in the case of non-marine plant and animal-derived bioactive peptides: elucidating correlation between the peptide structure and its effect and demonstrating its stability in vivo. The structures of marine bioactive peptides are highly variable and complex and dependent on the sources from which they are isolated. They can be cyclical, in the form of depsipeptides, and often contain secondary structures. Because of steric factors, marine-derived peptides can be resistant to proteolysis by gastrointestinal proteases, which presents an advantage over other peptide sources. Because of heterogeneity, amino acid sequences as well as preferred mechanisms of peptides showing specific bioactivities differ compared to their animal-derived counterparts. This review offers insights on the extreme diversity of bioactivities, effects, and structural features, analyzing 253 peptides, mainly from marine food sources. Similar to peptides in food of non-marine animal origin, a significant percentage (52.7%) of the examined sequences contain one or more proline residues, implying that proline might play a significant role in the stability of bioactive peptides. Additional problems with analyzing marine-derived bioactive peptides include their accessibility, extraction, and purification; this review considers the challenges and proposes possible solutions.

## 1. Introduction

Although marine peptides have only fairly recently garnered deserved attention (especially compared to peptides from other plant/animal sources), their potential to generate classes of peptides with interesting properties such as antiaging, antituberculosis, anticoagulant, and antidiabetic makes them promising agents not only in medicine and pharmacy [1,2,3], but also in the cosmetic industry [4,5,6]. Because of the beneficial interactions of the marine peptides with phenolic compounds [7], and to the improved emulsifying and foaming properties [8], their usage in the food industry has also proven to be valuable. Attempts have also been made to employ peptides derived from seaweed in prebiotics and nutraceuticals [9]. The use of marine waste for peptide generation is not only useful from economic and ecological standpoints, but can also produce peptides with proven ACE (angiotensin-converting enzyme) inhibitory, antioxidant, and immunomodulatory activity [10,11,12,13,14].

However, several problems regarding wide-spread use of marine-derived peptides still need to be solved: finding optimal conditions for the isolation of peptides from their sources and creating uniform conditions for their production from particular sources; establishing correlations between structure and bioactivity; demonstrating the peptide’s stability and effectiveness in vivo; and in certain cases, improving their accessibility and extraction yields. For example, in the case of marine peptides derived from seaweed, both the accessibility of source material and the inefficiency of peptide extraction present problems. Extraction of seaweed-derived peptides is additionally hindered by the presence of polysaccharides in cell walls of the seaweed [15].

Similarly to peptides of other animal and plant origin, the structures of marine bioactive peptides are highly dependent on the source from which they are isolated [16,17]. But such variations in activities and structures of marine peptides are even more pronounced because of the high taxonomic diversity among and within the five major groups of marine organisms used as food, fish, algae, bivalves, cephalopods, and crustaceans, spanning four kingdoms of living organisms.

However, unlike peptides from other sources, many marine-derived peptides are more resistant to proteolysis by gastrointestinal proteases, because of the steric factors derived from their unusual structures (branched, cyclic and possessing both D and L amino acids) [18,19].

Thus, the aim of this work is to present the current understanding of marine peptides’ production, structures, and applications, and also to compare and contrast them with production, structures, and applications of peptides from other animal food sources.

In order to achieve that, we comprised a list of 253 peptides from all five groups of marine organisms used as food (Table S1), using the BIOPEP database [20] and our own survey of literature. Reason why BIOPEP was chosen for peptide selection is two-fold: first, it is currently the most inclusive database, since it encompasses all bioactive peptides, regardless of their origin, effect, or length, with 4031 entries as of August 2020. Second, it provides additional information, such as in cases of ACE inhibitors, EC50 value, and type of organism from which peptide(s) were extracted. Although other databases for marine products exist, they are either too broad, such as the marine natural product database [21] or too narrow, focusing only on a specific type of organism and class of peptides (such as PenBase [22]) or a specific length (such as PepBank [23]) or a particular bioactivity [24,25,26,27,28,29]. Thus, we decided to use BIOPEP as the database most closely appropriate and add any missing sequences found during our literature survey. Our decision to include peptides from freshwater algae [15,16,17] in our analysis rests on the fact that a large number of them possess interesting medicinal potential (including peptides with very efficient antithrombotic and antidiabetic properties). Decision to include peptides from freshwater algae was also driven by two additional factors: the number of marine algae-derived peptides with known sequence is relatively low and freshwater algae are often used as food.

**Figure 1:**
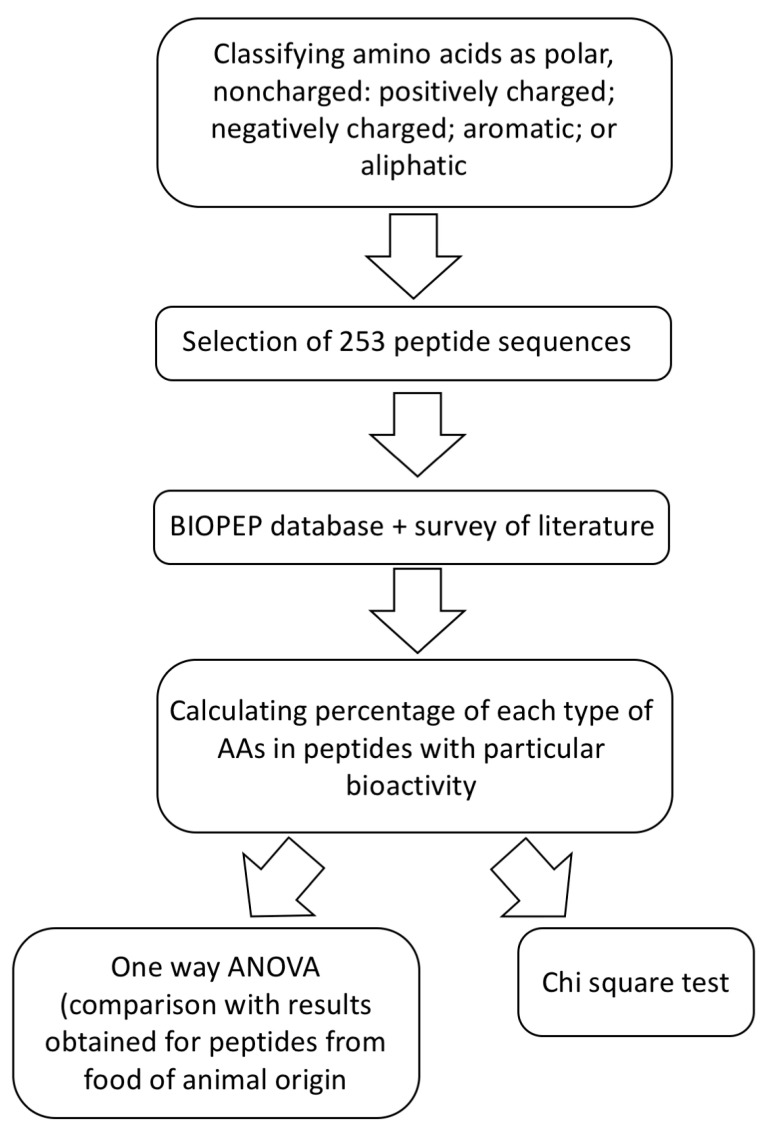
Flow chart for obtaining and analyzing data.

We included peptides ranging from 2 to 40 amino acid (AA) residues in length, reporting their sequence, the source from which they were isolated, their bioactive effect, and their EC50 value (where available) (Table S1). The complete set of peptides derives from eight different phyla, and represents 14 classes, from Eubacteria, Chromista, Plantae, and Animalia. To perform statistical analysis, we followed the flow chart given in Figure 1. First, we classified peptides according to their bioactivity (ACE inhibitors, antioxidative, immunomodulatory, antimicrobial, antithrombotic, and antidiabetic). Second, we classified AA residues, as in our previous paper [30], either as aliphatic (glycine (G) alanine (A), leucine (L), isoleucine (I), proline (P), and valine (V)); as aromatic (tryptophan (W), phenylalanine (F), and tyrosine (Y)); as polar noncharged (asparagine (N), glutamine (Q), methionine (M), cysteine (C), serine (S), and threonine (T)); as positively charged (histidine (H), arginine (R), and lysine (K)); as negatively charged (aspartic acid (D) and glutamic acid (E)). Third, we calculated the percentages of each class of amino acid present in peptides of a specific activity. Then, statistical tests were performed. One-way analysis of variance (one-way ANOVA) was used to assess if there is a significant influence of amino acid composition of peptide on its activity. The effect of the type of AA residue on peptide activity was assessed using the chi-square (χ^2^) test [31]. Additionally, we compared results for marine peptides with results for peptides from food of non-marine animal origin (taken from 30) to see if there are differences between sequences of peptides exhibiting the same effect in different types of food sources.

## 2. Isolation and Purification of Marine-Derived Peptides

**Figure 2:**
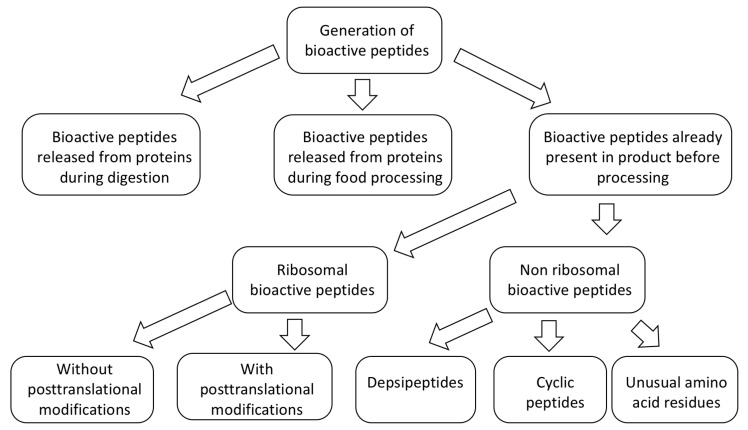
Generation of marine bioactive peptides.

The bioactive peptides may already be present in some food samples. They can also be generated either by processing (during preparation of hydrolyzates or technological operations) or released from parent protein during digestion (Figure 2). Peptides already present in products can be either ribosomal or non-ribosomal. Non-ribosomal peptides are those peptides that are synthesized by non-ribosomal peptide synthetase (NRPS) enzymes [32] rather than on ribosomes. Among the marine bioactive peptides, a significant number of the pharmacologically attractive and the most researched peptides are non-ribosomal. In marine organisms non-ribosomal peptides are present mostly in marine bacteria and sponges [33,34]. All peptides listed in Table S1 are of ribosomal origin.

As in the case of peptides from other animal and plant sources, isolation and production of marine-derived peptides face a few challenges. Such challenges primarily arise from (i) the lack of standardization of hydrolysis and/or extraction processes, (ii) usage of the whole hydrolyzate (instead of individual peptides) to determine activity, (iii) technological limitations in methods for purification and analysis, and (iv) as stated previously, variation in composition and primary structure of peptides between different groups of marine organisms.

Two major reasons could be identified as causes for hydrolysis standardization problems:

1. Usage of insufficiently characterized, crude enzyme preparations obtained from another organism [35,36,37]. Although the efficiency of this method of extraction might be high, the presence of large numbers of proteases with different specificities [38] can make reproducibility of the results questionable and hinder standardization of this extraction method.
2. Change in ratio of enzyme(s) to substrate, conditions of hydrolysis, and in ratio of proteases (if more than one protease is used) (Table 1).

Because of their different Km and specificities, such changes in enzyme/substrate ratios and/or temperature and pH will have a significant impact on the number and type of peptides produced [42,60,61,62].

Additionally, prior to hydrolysis, extraction is usually done by organic solvents, buffers, or water. The extract is further purified by application of often multiple types of chromatographic methods [66,67,68,69,70,71,72,73,74]. However, as with the often variable conditions for hydrolysis, extraction of marine peptides, even from the same class of organisms, is often done with buffers of variable compositions [72,73,74] or different combinations of organic solvents [75,76,77]. Such practice, although allowing the extraction of new peptides with different bioactivities, is adding to variability and complexity of the results.

Although hydrolyzate is usually further fractionated and individual peptides are separated, sometimes the extract as a whole is purified and analyzed further [42,67,69,70,78]. This prevents the assessment of individual peptides, and in turn the establishment of correlation between each peptide’s structure and its bioactivity.

Technological limitations present another challenge when it comes to correlating structure and bioactivity of individual peptides. Those technological limitations can be broadly classified as problems with separation of bioactive peptides and problems with their analysis. Most separations are performed using either ultrafiltration or chromatographic techniques that separate peptides based on their charge, size, or hydrophobicity. Considering that peptides are often of very similar size, charge, and/or hydrophobicity, and that most of these fractionation techniques take into account only one of the mentioned characteristics, isolation of individual peptides is hindered. Proposed solutions to this problem are two-fold: employment of orthogonal or multidimensional separation systems or inclusion of additional steps, such as electrodiffusion [79]. In regard to the challenges of analyzing individual peptides, which is usually done by mass spectrometry, a solution might be found in using more sophisticated analyzers such as ion traps or triple quadrupoles [80].

As stated before, the isolation of peptides from certain classes of marine organisms requires additional steps, such as the addition of enzymes (cellulase and/or xylanase) for extractions of peptides from marine algae [15].

Because of the great variety of marine organisms, it is hard to give recommendations for one-fits-all solution that can act for extraction/hydrolysis, purification, and identification of marine peptides. Especially since, as stated before, certain organisms require additional steps in procedure or specific conditions (e.g., disruption of cell wall in algae and bacteria, multiple washing steps during organic extraction of peptides from fish via surimi process [81], etc.). However, the main question that dictates both extraction/hydrolysis step as well as purification step is whether we are performing only an exploratory search (e.g., accessing all possible activities) or whether we are looking for peptides with specific activities. This is a question of crucial importance because it determines the selection of an appropriate method. For example, both extraction with organic solvents and extraction with acids/alkaline solutions (followed by isoelectric precipitation) can lead to the production of variety of peptides, but both of these methods are time-consuming, environmentally dangerous, and require longer purification process [81,82]. Additionally, because of the harsh condition and/or changes in pH both these methods can damage proteins and could result in loss of amino acids such as tryptophan [81,82]. Fermentation can also be applied for extraction of marine peptides [41], but depending on the strain(s) used and required experimental conditions (namely, temperature and pH) this can also lead to additional reactions of amino acids and changes in hydrophobicity [83]. Application of certain techniques, such as ultrasound-assisted extraction or microwave-assisted extraction was proven to result in high number of bioactive peptides, which was especially useful for extraction of peptides from algae, since it also leads to the rupture of cell wall [82,84]. One of the problems with those methods is variable yield [82,84]. Enzymatic hydrolysis is a highly specific and reproducible technique, but as stated before, because of the complexities of marine organisms and interactions of their proteins with other components within the cell, will not produce the same peptides in all marine organisms [15,41,84]. Additionally, as discussed above, hydrolysis with multiple proteases results simultaneously in high number of peptides with different bioactivities, while application of single proteases produces fewer peptides, but with similar effects. Another important factor to consider during enzymatic hydrolysis is the level of stability of peptides. Since, as would be discussed in following sections, the presence of proline enhances the stability of peptides, the usage of trypsin for hydrolysis might be beneficial.

As discussed, purification of extract/hydrolyzate is usually done by combination of filtration and chromatographic techniques. Such procedure also carries its challenges: ultrafiltration, which is commonly used to separate peptides from larger components in the extract/hydrolyzate, is relatively a cheap and fast method (especially if used in reactors), but usually requires several repetitions (because of decrease in selectivity and accumulation of peptides on membrane surface) and does not eliminate salts and other soluble components that can interfere with chromatographic separation. Certain improvements of filtration technique, such as application of electric field, pressure gradient, and/or electrodialysis cell, have been employed (with different level of success) [85]. Chromatographic techniques use characteristics such as polarity, charge, or affinity for separation of peptides. These methods all have several major drawbacks: first, the separation efficiency is impacted by number of peptides in the hydrolyzate; second, the separation condition (especially temperature) impacts greatly on both speed and efficiency of separation (which can greatly impact reproducibility of results); third, individual types of chromatography separate peptides based on only one characteristic, so multiple different chromatographic methods must be applied for separation of complex mixtures [79,86]. A potential solution for at least one of these drawbacks is the application of multi-dimensional liquid chromatography (MD-LC) in which peptides could be simultaneously separated based on two characteristics [86].

## 3. Comparison of the Structures of Marine-Sourced Peptides with Other Animal-Sourced Peptides and the Impacts of Protein Structure on Their Activity

Although different classifications of effects of bioactive peptides can be found in literature, the most often mentioned ones include: ACE inhibitors, antioxidative, antimicrobial, immunomodulatory, antithrombotic, and antidiabetic peptides. Table S2 summarizes the main bioactivities and mechanisms of actions. ACE inhibitors act as inhibitors for ACE, resulting in decrease of blood pressure. Antioxidative peptides help prevent or reduce damage caused by free radicals, using different mechanisms [30] (Table S2). Antimicrobial peptides prevent growth and reproduction of microorganisms. Immunomodulatory peptides help boost adaptive immune response, and are the most heterogenous group in terms of mechanisms employed [30]. Antithrombotic peptides help prevent platelet aggregation, thus decreasing the risk of diseases like stroke, arterial sclerosis, etc. Antidiabetic peptides help decrease the concentration of glucose in the bloodstream, also by using multiple mechanisms (Table S2).

**Figure 3:**
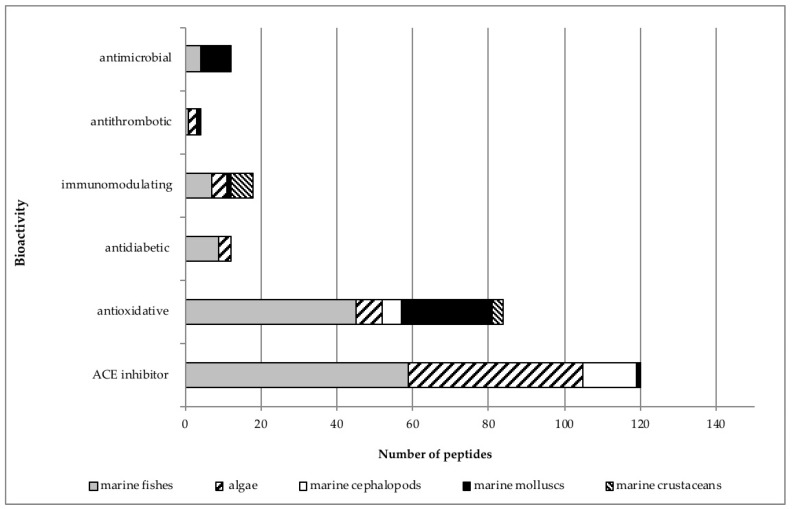
Distribution of bioactive peptides in the different groups of marine organisms. Peptides with multiple activities have been counted in each category.

As with the peptides of non-marine animal origin [30], the percentages of bioactive marine peptides isolated from different organisms vary depending on their effect (Figure 3). Over-representation of ACE inhibitors and antioxidative peptides in the literature can be partly explained by economic incentives driving research on drugs [87,88] to fight the rise of cardiovascular and antioxidant diseases [89,90].

Often peptides can be multifunctional, exhibiting more than one effect [91,92,93,94]. There are three important reasons for such occurrence: the peptides might act like signaling molecules at a systemic level [95], or there might be connections between metabolic pathways [93,94,96,97,98,99], or peptides may utilize more than one mode of action [30,100]. For peptides of certain bioactivity, such as antioxidative or antithrombotic peptides, the mode of action is well-known and relatively simple [30,56,101,102] (Table S2). On the other hand, for some categories, such as immunomodulatory peptides, different modes of actions are exhibited simultaneously and to a different extent in different source organisms [103,104,105,106,107] (Table S2). Variability and complexity of marine peptides has slowed the elucidation of the mechanism behind some activities. More intriguingly, several of the proposed mechanisms for marine-derived peptide activities are quite unique and different compared to other animal-derived peptides [30]. Examples include the interaction between cytotoxic peptides from sea anemones and voltage-dependent membrane channels [108], and the antimalarial effect of C Phycocyanin (isolated from marine cyanobacteria), exhibited through binding to ferriprotoporphyrin IX [2]. These findings could show that either specificities among organism classes dictate the particular mode of action, or that the structure of the peptides themselves is different.

Given these different modes of actions, different tests are used to determine specific effects of a given peptide (Table S2). This fact is especially problematic when it comes to immunomodulatory peptides, since if all aspects of immune response (cytotoxicity, activation of macrophages and neutrophils, up-regulation of caspase genes and propagation of apoptosis, etc.) were to be tested that would be both time consuming and highly costly. Additionally, even when testing for activities, such as antimicrobial, ACE inhibitory or antioxidative, for which few, well-defined assays are commonly employed, such assays often have different detection limits, specificity, reproducibility, and/or show preferential detection of compounds with given characteristic (such as polarity in the case of antioxidative tests) (Table S2). A further point of confusion is that different tests use different units to demonstrate effect (Table S2), thus making difficult any comparison of activity of different peptides. Therefore, efforts should be made to define which test should be used to monitor specific effects (and consequently in which units effects are measured) in order to obtain clear insight into peptides bioactivities.

**Figure 4:**
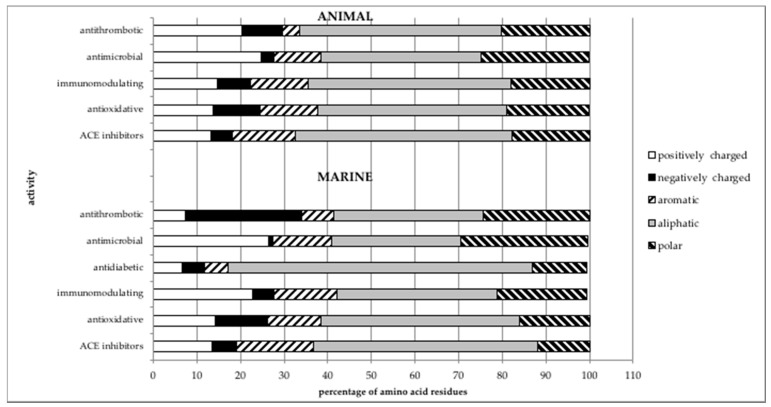
Comparison of amino acid distribution in bioactive peptides from different sources. Bioactive peptides from animal (non-marine) sources have been described in Maestri et al. 2019 [30]. Bioactive peptides from marine organisms are described in this paper. The five main categories of amino acids are as described in the text. Deviation from 100% can be explained by the presence of multifunctional peptides, unusual amino acid residues, etc.

As expected, the percentage of AA residue types in marine-derived peptides varies depending on the peptides’ bioactivity (Figure 4). To confirm this, we compared observed and expected frequencies for each type of AA residues (aliphatic, aromatic, noncharged polar, and positively and negatively charged) using the χ^2^ test. As in our previous paper [30], the expected frequencies were calculated using the following formula:

Expected frequency = (the number of amino acids in a given group × the total number of peptides with a particular effect)/20

The χ^2^ test [31] showed significant differences for observed and expected frequencies of AA residues in specific classes of bioactive peptides (χ^2^ (1,8) = 2150.07; *p* = 2.52 × 10^−15^).

Difference in percentages of different AA residues in peptides with particular bioactivity can be explained through correlations between structure and function. For example, a high percentage of hydrophobic (aliphatic and aromatic) AA residues in antidiabetic peptides (Figure 4) [109,110,111] is necessary for inhibition of dipeptidyl peptidase IV, a key enzyme involved in degradation of incretins [110,112,113], and for recognition of receptors for glucagon-like peptides [114,115]. A high percentage of negatively charged AA residues (Figure 4) has been reported in antithrombotic peptides showing both direct thrombin inhibition [116,117] and inhibition of factors in intrinsic pathways [72,118]. A high frequency of positively charged AA residues in so-called cationic antimicrobial peptides (Figure 4) [119,120,121] is proven to be necessary for interactions with anionic microbe membranes.

Similar to the results obtained for non-marine animal peptides [30], a significant percentage (52.7%) of examined sequences contain one or more proline residues (Table S1). This could imply that proline plays a significant role in peptide stability [18]. Therefore, the use of trypsin to generate stable peptides might be a valuable consideration [122].

When data for percentage of AA residues per category for animal-derived peptides described in our previous work [30] and marine-derived peptides (Figure 4) are compared, dependency of peptide’s structure from source becomes visible and suggestive of possible peculiarities. To estimate these differences, we used the one-way ANOVA test to compare the percentages of polar noncharged, aliphatic, aromatic, and positively and negatively charged AA residues per activity (antithrombotic, antimicrobial, immunomodulating, antioxidative, and ACE inhibitors) in non-marine animal and marine sources. As previously stated, the source organisms for the marine peptides are taxonomically diversified, ranging from bacteria to plants and animals. Antidiabetic marine-derived peptides were not included because of the insufficient data for antidiabetic peptides from other animal sources. Results of one-way ANOVA showed that for each bioactivity, with the exception of the antithrombotic peptides, the percentages of AA residues of a given category were statistically different at *p* = 0.05 (ACE inhibitors: F(4,5) = 137.26; *p* = 2.69 × 10^−5^; antioxidative: F(4,5) = 253.87; *p* = 5.86 × 10^−6^; antimicrobial: F(4,5) = 36.32; *p* = 0.00069; immunomodulatory: F(4,5) = 18.40; *p* = 0.0034; antithrombotic: F(4,5) = 5.1; *p* = 0.052).

There are three possible explanations for such occurrence. First, as previously stated, marine peptides come from taxonomically diverse groups of highly specific organisms, and this can have great impact on both peptide structure and function: the sample of peptides of animal origin described in [30] derives from few species in the phylum Chordata, comprised of classes Aves and Mammalia. On the contrary, the sample of marine peptides encompasses 14 different classes in 4 kingdoms. Additionally, even if not considered in this analysis, this is especially prominent in the case of peptides isolated from sponges and mollusks, which can be cyclical or composed of “unusual” amino acid residues [19,41,84,123,124,125]. Second, this opens the possibility that other mechanisms exist beyond those found and described in non-marine-derived peptides [30]. For example, Kouno et al. [126] found that dried bonito hydrolyzate contains peptides that regulate in vivo blood pressure by directly acting on vascular smooth muscle, instead of acting through ACE inhibition. Additionally, certain marine-derived antimicrobial peptides can also act as K^+^ blockers [127] (Table S2). However, further studies showing the modes of action of different classes of marine peptides are needed. Third, as shown in Figure 3, distribution of peptides showing specific bioactivities is not uniform across all marine organisms, with a prevalence of fish, algae, and bivalves. Moreover, some categories of peptides are derived from few species (see Figure 3 and Table S1).

Because of the above mentioned factors, combined with overall differences in the literature concerning marine-derived vs. animal-derived peptides, comparisons between specific groups (for example terrestrial Chordata vs. fish) are not possible at this point. Hence, in this analysis, we chose to compare all animal-derived peptides showing particular bioactivity with all marine-derived peptides exhibiting the same effect. As stated, results of such comparison would be skewed and should be taken with caution.

Further point to be taken into account when discussing amino acid composition of peptides showing a specific effect is whether any indication exists that the mechanism of action of the marine peptide depends on the type of organism from which it is isolated [105]. Since, as we discussed, difference in preferred mechanism stems from difference in amino acid composition, this implies that inclusion of all groups of marine organisms into analysis can skew the results. For example, peptides from fish showing both antimicrobial and immunomodulatory activities, as well as immunomodulatory peptides from crustaceans, are rich in positively charged and polar amino acids (Figure 4). High percentage of polar and positively charged amino residues in marine organisms can be a consequence of certain specificities of their mechanisms of action, such as interaction with antibodies and complement system, prevention of formation of biofilms, induction of cytokine synthesis, suppression of NO production (Table S2) [105,128,129,130]. Since the majority of antimicrobial peptides included in this analysis are isolated from fish and bivalves (Figure 3) this could be an additional factor accounting for a large difference in content of positively charged and polar amino acids among antimicrobial peptides from marine and animal sources. Given that ACE inhibitors isolated from marine fish and algae (that made up the biggest portion of ACE inhibitory peptides in this analysis; Figure 3) also show non-competitive inhibition as a mechanism of action [131,132] this could explain a slightly higher ratio of aromatic and aliphatic amino acid residues in peptides isolated from marine sources compared to animal sources. The majority of antioxidative peptides in this analysis was isolated from marine fish and bivalves (Figure 3). Slightly higher ratio of positively and negatively charged amino acid residues in antioxidative peptides of marine origin compared to antioxidative peptides of animal origin (in which higher ratio of polar amino residues was observed, [30]) is harder to explain, since seemingly they do not show differences in mechanism of action (Table S2). One possible explanation for such discrepancy could be overrepresentation of peptides from certain marine species in the literature and in this analysis.

## 4. Stability of Marine-Derived Peptides In Vivo

The stability of peptides in vivo is determined by several factors, most importantly the peptides’ size and structure [30,133]. Besides influencing the speed of a peptide’s transport from lumen to enterocytes, the size of the peptide will also influence the mode of transport (paracellular, transcellular or via peptide transporters-PEPT1 and PEPT2) [134,135,136]. The type of amino acid residues, as well as the shape of the peptide, greatly influences its susceptibility to degradation by gastrointestinal proteases [137,138]. This could explain the high percentage of proline residues in examined sequences, since the presence of proline promotes increased stability toward protease degradation [18,139,140,141].

Marine-derived peptides have several other characteristics as well that can increase their in vivo stability compared to other animal and plant sourced peptides. These properties contribute to “unusual” structures and make peptides less likely to be recognized by digestive enzymes, thus preventing the formation of enzyme-peptide complex. These characteristics can be broadly classified as the following:

1. High percentage of proline and branched amino acid residues [18,19,142];
2. Presence of both d and l amino acids [19,84,143]
3. Cyclic structure [18,19,123,124,125,144,145,146,147]. Steric factors are the reason why cyclic peptides are more resistant to digestion, because of their cyclic nature neither N nor C terminus can be recognized by proteases (either during processing or in gastrointestinal tract) [148,149,150].
4. Peptides in form of depsipeptides. A depsipeptide is one in which one or more amide bonds are substituted with ester bonds [151,152,153], often leading to the formation of cyclic depsipeptides [154,155,156,157,158].
5. Presence of unusual AA residues, such as bromotryptophan [84,159,160]. Bromotryptophan, as brominated tryptophan, is non-coded amino acid that is usually found in non-ribosomal proteins and peptides, although some ribosomal proteins of marine origin were shown to contain 6-bromo-l-tryptophan [160,161].
6. Presence of secondary structures [41,127,162,163,164,165]. Given that formation of α helix requires only 4 amino acid residues per turn it can be formed in shorter peptides. Therefore, α helix is the most common secondary structure in marine peptides, although in longer peptides β sheets can also be present [163].

Not all groups of marine organisms produce peptides with all of these characteristics, and some of these characteristics are more common in certain groups. Cyclic peptides are often found in marine sponges and mollusks [19,84,138,145,146], while cyclic depsipeptides are often isolated from marine bacteria [153,155,157,158]. Secondary structures, especially α helix, are common in antimicrobial marine peptides [162,163,164,165], because the presence of α helix allows penetration through a microbe’s membrane [163,164].

However, although all these factors contribute to the peptides’ stability in vivo, bioactivities are usually tested during in vitro experiments. Correlating results obtained in in vitro experiments with the conditions existing in the human gastrointestinal tract presents a major challenge in determining the in vivo stability. Certain factors that can influence both the stability and the digestibility of peptides in vivo are often not considered during in vitro generation. These factors include: Using enzymes that are very different from digestive enzymes in their structures/activities or using them in ratios different than physiological ones; not accounting for the effects of technological processing (effects of temperature, pressure or fermentation); not considering changes in pH between different parts of the gastrointestinal tract; not analyzing the “matrix effect”(the interaction with other components in food or the body); and not counting the effect of peristaltics. Some attempts to use gastrointestinal proteases for peptide generation in the same ratio present in the human duodenum have been made [166,167]. But changes in pH, the “matrix effect” and the effect of peristaltics still cannot be properly assessed, especially with the static digestion models that are commonly being employed.

Fortunately, because of the significant number of antitumor, antidiabetic, and antihypertensive peptides isolated from marine sources [41,84,168,169,170,171,172,173,174], epidemiological studies and clinical trials are now under way. Therefore, at least for these types of peptides, the relative influence of different factors affecting peptides’ activity and correlation between in vitro and in vivo effects will be determined in the near future.

An additional point should be considered here: increased stability of a peptide diminishes its digestibility. Decreased digestibility, although important in preserving peptide’s activity, can lead to allergic reactions [175,176].

## 5. Usage of Whole Hydrolyzate vs. Purified Peptides vs. Synthetic Peptides in Production of Food and Marine Drugs

Marine peptides and hydrolyzates can be utilized in two principal ways: as food/feed supplements and as drugs [2,41,82,84,177,178,179,180,181,182,183,184,185,186,187]. Whole hydrolyzates of marine-derived proteins are usually applied in food/feed preparations, where they serve different functions, as antioxidants [178], hypolipidemic agents [181,182], enhancing physical performance in elderly people [177], and preventing hair loss [180]. Although all these activities have been verified by clinical trials, there are several problems with using whole protein hydrolyzates. First, the composition of the hydrolyzates (the type and content of individual amino acids) is greatly dependent on the type and ratio of enzymes used for hydrolysis as well as on the pH during hydrolysis [188,189]. Second, in whole hydrolyzates it is hard to determine which peptides are biologically active. Third, because of complex compositions, it becomes hard to predict their behaviors in vivo. However, there are two significant advantages in using whole hydrolyzates: cost and time. The methods and chemicals necessary to isolate and characterize individual peptides (such as the use of chromatographic techniques and mass spectrometry) both prolong and add expenses to the creation of products able to be tested for in vivo activity. Research to improve the extraction step can enhance the number of peptides with more pronounced and/or different bioactivities in hydrolyzate. For example, microwave assisted extraction [82] has proven to be a good method for enhancing peptide’s bioactivity.

Individual peptides require identification, purification, and analysis before being used in food preparation or drugs. Therefore, their cost is higher compared to hydrolyzates. This is a particular problem with antimicrobial peptides that are more expensive to produce than “commercial” antibiotics [84], and thus a less attractive option. Another problem is when the mode of action of an individual peptide is unknown [183,185], as it leads to unexpected results due to metabolic pathway interconnections [95]. Such is the case with antidiabetic peptides from salmon skin which, although they caused decrease in blood level glucose, also increased levels of HDL (high-density lipoproteins) [171]. Furthermore, certain peptides exhibit their effect preferentially in specific tissues [190]. Because of the “matrix effect” and problematic correlations with animal models, peptides that showed particular bioactivity in vitro or in animal models can show variable results in clinical trials [41]. Since such variations in activity can be partially ascribed to decreased stability, encapsulation and the use of different carriers are methods being considered and employed [191,192,193,194]. Conversely, these methods can lead to difficulty in a peptide’s release and therefore hinder its activity [193,194].

Because of the prevalence of cancer and diabetes mellitus type 2, antidiabetic and anticancer peptides are the most interesting targets for synthesis [41,84,183,184,185]. Since most of the peptides exhibiting anticancer effects have cyclical structures [29,84,145,154,167,170,173,174], this makes production of synthetic anticancer peptides difficult. Also, as said previously, although the stability of such cyclic peptides is high, their digestibility is lower than linear peptides. This can prevent their transport into enterocytes and from enterocytes into capillaries, thus hindering their activity.

In all of these cases, one important point has not received the attention it deserves. A large number of in vivo studies are still performed on rats and mice [36,62,84,126,169,171,181]. Given the clear differences in anatomy and physiology, correlation between animal models and humans is questionable [195,196]. Additionally, since age, health, nutrition, and hormonal status [197,198,199] of the subject can all influence the peptides’/hydrolyzates’ effects, precaution should be taken during selection of subjects for clinical trials.

## 6. Conclusions

Although there are still many challenges to be solved, mainly harmonization of extraction methods and hydrolysis, as well as correlation between in vitro and in vivo results, marine organisms have proven to be invaluable sources of peptides with unique structures and diverse bioactivities. Because marine peptide research has only recently gotten its fair share of attention, many of their mechanisms of action are still unknown. In future, as more marine-derived peptides are isolated and characterized, comparisons of structures and activities with counterparts from other organisms will become more informative. However, the construction of databases, such as StraPep [200] (http://isyslab.info/StraPep/, accessed August 2020), that correlate the structures of bioactive peptides with their functions will help solve this problem. Additionally, correlating structures with mechanism of action will help not only with constructing proper peptide carriers and predicting their release and possible undesired effects in clinical trials, but can also provide useful guidance in searching for peptides with the same potential.

## Figures and Tables

**Table 1:** Commonly used proteases for production of marine-derived peptides.

Protease/Proteases Combination	References
Alcalase	[39,40,41]
Bromelain	[7,42,43]
Proteinase K + thermolysin	[44,45,46]
Thermolysin	[47,48,49,50]
Pepsin	[51,52,53]
Proteases	[54,55,56]
Alcalase + pepsin + chymotrypsin	[57,58,59]
Neutrase	[53,60,61]
Papain	[62,63,64]
Neutral protease + papain	[65]

## Supplementary Materials

The following are available online at https://www.mdpi.com/1660-3397/18/8/424/s1: Table S1: Sequences, activity, source material, EC50 (where applicable), and length of the 253 marine-derived peptides used for analysis in this paper. Table S2: Description of main bioactivities of peptides, mechanisms of action and bioassays for testing.
