# Supplementary material for: Marine Bioactive Peptides—An Overview of Generation, Structure and Application with a Focus on Food Sources
Source: Mar Drugs. 2020 Aug 13;18(8):424. doi: 10.3390/md18080424 (PMC7460072; doi:10.3390/md18080424)
Supplement: Supplementary file 1 [file marinedrugs-18-00424-s001.pdf]

marine drugs

MDPI

Review

Marine Bioactive Peptides—An Overview of Generation, Structure, and Application with a Focus on Food Sources

Milica Pavlicevic [1], Elena Maestri [2,3,*] and Marta Marmiroli [2]

1 Institute for Food Technology and Biochemistry, Faculty of Agriculture, University of Belgrade, 11070 Belgrade, Serbia; mpavlicevic@agrif.bg.ac.rs
2 Department of Chemistry, Life Sciences and Environmental Sustainability, and SITEIA.PARMA, University of Parma, 43124 Parma, Italy; marta.marmiroli@unipr.it
3 Consorzio Italbiotec, Via Fantoli 16/15, 20138 Milan, Italy
* Correspondence: elena.maestri@unipr.it

Table S1. – Complete list of peptides from marine organisms and corresponding references.

Sequence	Activity	Source	Taxonomy*	EC50	Length	Note	Reference
AW	antioxidative	marine Bivalvia	*Mactra quadrangularis* Reeve	/	2	Whole protein hydrolyzate; ribosomal	[1]
CF	ACE inhibitor	marine Pisces	Elasmobranchii	1.96	2	Whole protein hydrolyzate; ribosomal	[2]
DI	ACE inhibitor	marine Pisces	*Scorpaena notata* Rafinesque	0.98	2	Whole protein hydrolyzate; ribosomal	[3]
EI	ACE inhibitor	marine Pisces	*Scorpaena notata* Rafinesque	1.69	2	Whole protein hydrolyzate; ribosomal	[3]
EY	ACE inhibitor	marine Pisces	Elasmobranchii	2.68	2	Whole protein hydrolyzate; ribosomal	[2]

FI	ACE inhibitor	marine Pisces	*Scorpaena notata* Rafinesque	1.44	2	Whole protein hydrolyzate; ribosomal	[3]
FY	ACE inhibitor	marine algae	*Undaria pinnatifida* (Harvey) Suringar	18.8	2	Whole protein hydrolyzate; ribosomal	[4]
GP	ACE inhibitor	marine Pisces	*Pollachius pollachius* Linnaeus	252.63	2	Whole protein hydrolyzate; ribosomal	[5]
IY	ACE inhibitor	marine Pisces	*Sardina pilchardus* Walbaum	2.1	2	Whole protein hydrolyzate; ribosomal	[6]
IY	ACE inhibitor	marine algae	*Undaria pinnatifida* (Harvey) Suringar	42.3	2	Whole protein hydrolyzate; ribosomal	[4]
KD	antioxidative	marine Pisces	*Katsuwonus pelamis* Linnaeus	/	2	Whole protein hydrolyzate; ribosomal	[7]
KL	ACE inhibitor	marine algae	*Undaria pinnatifida* (Harvey) Suringar	50.2	2	Whole protein hydrolyzate; ribosomal	[8]
KP	ACE inhibitor	marine Pisces	*Katsuwonus pelamis* Linnaeus	22	2	Whole protein hydrolyzate; ribosomal	[9]
KW	ACE inhibitor	marine Pisces	*Sardina pilchardus* Walbaum	1.63	2	Whole protein hydrolyzate; ribosomal	[6]
KY	ACE inhibitor	marine algae	*Undaria pinnatifida* (Harvey) Suringar	61	2	Whole protein hydrolyzate; ribosomal	[4]
LW	antioxidative	marine Bivalvia	*Mactra quadrangularis* Reeve	/	2	Whole protein hydrolyzate; ribosomal	[1]

LY	ACE inhibitor	marine Pisces	*Sardina pilchardus* Walbaum	18	2	Whole protein hydrolyzate; ribosomal	[6]
MF	ACE inhibitor	marine Pisces	*Sardina pilchardus* Walbaum	45	2	Whole protein hydrolyzate; ribosomal	[6]
MY	ACE inhibitor	marine Pisces	*Sardina pilchardus* Walbaum	193	2	Whole protein hydrolyzate; ribosomal	[6]
MY	antioxidative	marine Pisces	*Sardina pilchardus* Walbaum	/	2	Whole protein hydrolyzate; ribosomal	[10]
NK	ACE inhibitor	marine algae	*Undaria pinnatifida* (Harvey) Suringar	810	2	Whole protein hydrolyzate; ribosomal	[8]
PL	ACE inhibitor	marine Pisces	*Pollachius pollachius* Linnaeus	337.32	2	Whole protein hydrolyzate; ribosomal	[5]
RY	ACE inhibitor	marine Pisces	*Sardina pilchardus* Walbaum	10.5	2	Whole protein hydrolyzate; ribosomal	[6]
TW	antioxidative	marine Bivalvia	*Mactra quadrangularis* Reeve	/	2	Whole protein hydrolyzate; ribosomal	[1]
VF	ACE inhibitor	marine Pisces	*Sardina pilchardus* Walbaum	9.2	2	Whole protein hydrolyzate; ribosomal	[6]
VW	antioxidative	marine Bivalvia	*Mactra quadrangularis* Reeve	/	2	Whole protein hydrolyzate; ribosomal	[1]
YH	ACE inhibitor	marine algae	*Undaria pinnatifida* (Harvey) Suringar	35.2	2	Whole protein hydrolyzate; ribosomal	[4]

YK	ACE inhibitor	marine algae	*Undaria pinnatifida* (Harvey) Suringar	610	2	Whole protein hydrolyzate; ribosomal	[8]
AEL	ACE inhibitor	freshwater algae	*Chlorella vulgaris* Beijerinck	/	3	Whole protein hydrolyzate; ribosomal	[11]
AFL	ACE inhibitor	freshwater algae	*Chlorella vulgaris* Beijerinck	/	3	Whole protein hydrolyzate; ribosomal	[11]
AKK	ACE inhibitor	marine Pisces	*Sardina pilchardus* Walbaum	3.2	3	Whole protein hydrolyzate; ribosomal	[6]
EAK	antioxidative	marine Pisces	*Katsuwonus pelamis* Linnaeus	/	3	Whole protein hydrolyzate; ribosomal	[7]
FAL	ACE inhibitor	freshwater algae	*Chlorella vulgaris* Beijerinck	26.3	3	Whole protein hydrolyzate; ribosomal	[12]
FAL	ACE inhibitor	freshwater algae	*Chlorella vulgaris* Beijerinck	/	3	Whole protein hydrolyzate; ribosomal	[11]
FKK	antioxidative	marine Crustacea	*Penaeus japonicus* Spence Bate	/	3	Whole protein hydrolyzate; ribosomal	[13]
FQP	ACE inhibitor	marine Pisces	*Katsuwonus pelamis* Linnaeus	/	3	Whole protein hydrolyzate; ribosomal	[14]
GAH	antioxidative	marine Pisces	*Sardinella aurita* Valenciennes	/	3	Whole protein hydrolyzate; ribosomal	[15]
GGE	antioxidative	marine Pisces	*Sardinella aurita* Valenciennes	/	3	Whole protein hydrolyzate; ribosomal	[15]

GHF	ACE inhibitor	marine Pisces	*Katsuwonus pelamis* Linnaeus	1001	3	Whole protein hydrolyzate; ribosomal	[8]
GLP	ACE inhibitor	marine Pisces	*Pollachius pollachius* Linnaeus	1.62	3	Whole protein hydrolyzate; ribosomal	[5]
GPL	ACE inhibitor	marine Pisces	*Pollachius pollachius* Linnaeus	2.65	3	Whole protein hydrolyzate; ribosomal	[5]
GRP	ACE inhibitor	marine Pisces	*Sardina pilchardus* Walbaum	19.9	3	Whole protein hydrolyzate; ribosomal	[6]
IAE	ACE inhibitor	freshwater algae	*Chlorella vulgaris* Beijerinck	34.7	3	Whole protein hydrolyzate; ribosomal	[12]
IAE	ACE inhibitor	freshwater algae	*Chlorella vulgaris* Beijerinck	/	3	Whole protein hydrolyzate; ribosomal	[11]
IFG	ACE inhibitor	marine Pisces	*Thunnus thynnus* Linnaeus	1001	3	Whole protein hydrolyzate; ribosomal	[8]
IKK	antioxidative	marine Crustacea	*Penaeus japonicus* Spence Bate	/	3	Whole protein hydrolyzate; ribosomal	[13]
IKL	antioxidative	marine Pisces	*Katsuwonus pelamis* Linnaeus	/	3	Whole protein hydrolyzate; ribosomal	[7]
IKP	ACE inhibitor	marine Pisces	*Katsuwonus pelamis* Linnaeus	6.9	3	Whole protein hydrolyzate; ribosomal	[14]
IYK	ACE inhibitor	marine algae	*Undaria pinnatifida* (Harvey) Suringar	177	3	Whole protein hydrolyzate; ribosomal	[8]

KAI	antioxidative	marine Pisces	*Katsuwonus pelamis* Linnaeus	/	3	Whole protein hydrolyzate; ribosomal	[7]
KFY	ACE inhibitor	marine algae	*Undaria pinnatifida* (Harvey) Suringar	45	3	Whole protein hydrolyzate; ribosomal	[8]
KVI	antioxidative	marine Pisces	*Katsuwonus pelamis* Linnaeus	/	3	Whole protein hydrolyzate; ribosomal	[7]
KYY	ACE inhibitor	marine algae	*Undaria pinnatifida* (Harvey) Suringar	79	3	Whole protein hydrolyzate; ribosomal	[8]
LDY	ACE inhibitor	marine algae	*Palmaria palmata* (Linnaeus) F.Weber & D.Mohr	6.1	3	Whole protein hydrolyzate; ribosomal	[16]
LDY	antioxidative	marine Bivalvia	*Mactra quadrangularis* Reeve	/	3	Whole protein hydrolyzate; ribosomal	[1]
LEQ	ACE inhibitor	marine algae	*Nannochloropsis oculata* (Droop) D.J.Hibberd	173	3	Whole protein hydrolyzate; ribosomal	[17]
LGP	ACE inhibitor	marine Pisces	*Pollachius pollachius* Linnaeus	0.72	3	Whole protein hydrolyzate; ribosomal	[5]
LHY	antioxidative	marine Pisces	*Sardinella aurita* Valenciennes	/	3	Whole protein hydrolyzate; ribosomal	[15]
LKL	ACE inhibitor	marine Pisces	*Sardina pilchardus* Walbaum	188	3	Whole protein hydrolyzate; ribosomal	[18]
LPG	ACE inhibitor	marine Pisces	*Pollachius pollachius* Linnaeus	5.73	3	Whole protein hydrolyzate; ribosomal	[5]

LRY	ACE inhibitor	marine algae	*Pyropia yezoensis* (Ueda) M.S.Hwang & H.G.Choi	5.1	3	Whole protein hydrolyzate; ribosomal	[8]
LRY	ACE inhibitor	marine algae	*Palmaria palmata* (Linnaeus) F.Weber & D.Mohr	0.04	3	Whole protein hydrolyzate; ribosomal	[16]
LTF	ACE inhibitor	marine Pisces	*Thunnus thynnus* Linnaeus	330	3	Whole protein hydrolyzate; ribosomal	[8]
LVE	ACE inhibitor	marine Bivalvia	*Pinctada fucata* Gould	14.2	3	Whole protein hydrolyzate; ribosomal	[19]
MKY	ACE inhibitor	marine algae	*Pyropia yezoensis* (Ueda) M.S.Hwang & H.G.Choi	7.3	3	Whole protein hydrolyzate; ribosomal	[8]
NHK	antioxidative	marine Bivalvia	*Mactra quadrangularis* Reeve	/	3	Whole protein hydrolyzate; ribosomal	[1]
NKL	ACE inhibitor	marine algae	*Undaria pinnatifida* (Harvey) Suringar	88	3	Whole protein hydrolyzate; ribosomal	[8]
PGL	ACE inhibitor	marine Pisces	*Pollachius pollachius* Linnaeus	13.93	3	Whole protein hydrolyzate; ribosomal	[5]
PLG	ACE inhibitor	marine Pisces	*Pollachius pollachius* Linnaeus	4.74	3	Whole protein hydrolyzate; ribosomal	[5]
RFH	ACE inhibitor	marine Pisces	*Pollachius pollachius* Linnaeus	331	3	Whole protein hydrolyzate; ribosomal	[6]
TDY	antioxidative	marine Bivalvia	*Mactra quadrangularis* Reeve	/	3	Whole protein hydrolyzate; ribosomal	[1]

VKL	antioxidative	marine Pisces	*Katsuwonus pelamis* Linnaeus	/	3	Whole protein hydrolyzate; ribosomal	[7]
VKV	antioxidative	marine Pisces	*Katsuwonus pelamis* Linnaeus	/	3	Whole protein hydrolyzate; ribosomal	[7]
YKY	ACE inhibitor	marine algae	*Undaria pinnatifida* (Harvey) Suringar	43.5	3	Whole protein hydrolyzate; ribosomal	[8]
YNK	ACE inhibitor	marine algae	*Undaria pinnatifida* (Harvey) Suringar	125	3	Whole protein hydrolyzate; ribosomal	[8]
AEVG	antioxidative	marine Pisces	*Mustelus griseus* Pietschmann	/	4	Whole protein hydrolyzate; ribosomal	[20]
AGSP	ACE inhibitor	marine Cephalopoda	*Sepia officinalis* Linnaeus	37.2	4	Whole protein hydrolyzate; ribosomal	[21]
AGSS	ACE inhibitor	marine Cephalopoda	*Sepia officinalis* Linnaeus	672.1	4	Whole protein hydrolyzate; ribosomal	[21]
AIYK	ACE inhibitor	marine algae	*Undaria pinnatifida* (Harvey) Suringar	810	4	Whole protein hydrolyzate; ribosomal	[8]
FIKK	antioxidative	marine Crustacea	*Penaeus japonicus* Spence Bate	/	4	Whole protein hydrolyzate; ribosomal	[13]
GAWA	antioxidative	marine Pisces	*Sardinella aurita* Valenciennes	/	4	Whole protein hydrolyzate; ribosomal	[15]
GDAP	ACE inhibitor	marine Cephalopoda	*Sepia officinalis* Linnaeus	22.5	4	Whole protein hydrolyzate; ribosomal	[21]

GWAP	ACE inhibitor	marine Pisces	*Sardinella aurita* Valenciennes	3.86	4	Whole protein hydrolyzate; ribosomal	[15]
IAPG	ACE inhibitor	freshwater algae	*Chlorella vulgaris* Beijerinck	11.4	4	Whole protein hydrolyzate; ribosomal	[12]
IAPG	ACE inhibitor	marine algae	*Arthrospira platensis* Gomont	11.4	4	Whole protein hydrolyzate; ribosomal	[12]
IAPG	ACE inhibitor	freshwater algae	*Chlorella vulgaris* Beijerinck	11.4	4	Whole protein hydrolyzate; ribosomal	[22]
ILAP	antidiabetic	marine algae	*Palmaria palmata* (Linnaeus) F.Weber & D.Mohr	43.46	4	Whole protein hydrolyzate; ribosomal	[23]
IVVE	ACE inhibitor	freshwater algae	*Chlorella vulgaris* Beijerinck	315.3	4	Whole protein hydrolyzate; ribosomal	[22]
KFYG	ACE inhibitor	marine algae	*Undaria pinnatifida* (Harvey) Suringar	610	4	Whole protein hydrolyzate; ribosomal	[8]
KFYG	ACE inhibitor	marine algae	*Undaria pinnatifida* (Harvey) Suringar	21	4	Whole protein hydrolyzate; ribosomal	[24]
LARL	antioxidative	marine Pisces	*Sardinella aurita* Valenciennes	/	4	Whole protein hydrolyzate; ribosomal	[15]
LLAP	antidiabetic	marine algae	*Palmaria palmata* (Linnaeus) F.Weber & D.Mohr	53.67	4	Whole protein hydrolyzate; ribosomal	[16]
LVSK	antioxidative	marine Bivalvia	*Mactra quadrangularis* Reeve	/	4	Whole protein hydrolyzate; ribosomal	[1]

MEMK	antioxidative	marine Bivalvia	*Mactra quadrangularis* Reeve	/	4	Whole protein hydrolyzate; ribosomal	[1]
MPDW	immunomodulating	marine algae	*Nannochloropsis oculata* (Droop) D.J.Hibberd	/	4	Whole protein hydrolyzate; ribosomal	[25]
NIGK	antithrombotic	marine algae	*Palmaria palmata* (Linnaeus) F.Weber & D.Mohr	/	4	Whole protein hydrolyzate; ribosomal	[7]
PAGE	antioxidative	marine Pisces	*Thunnus thynnus* Linnaeus	/	4	Whole protein hydrolyzate; ribosomal	[26]
PHYL	antioxidative	marine Pisces	*Sardinella aurita* Valenciennes	/	4	Whole protein hydrolyzate; ribosomal	[15]
PKAV	antioxidative	marine Pisces	*Katsuwonus pelamis* Linnaeus	/	4	Whole protein hydrolyzate; ribosomal	[7]
VDYP	antioxidative	marine Pisces	*Thunnus albacares* Bonnaterre	/	4	Whole protein hydrolyzate; ribosomal	[26]
VEGY	ACE inhibitor	freshwater algae	*Chlorella vulgaris* Beijerinck	128.4	4	Whole protein hydrolyzate; ribosomal	[22]
VGEY	ACE inhibitor	freshwater algae	*Chlorella ellipsoidea* Gerneck	128.4	4	Whole protein hydrolyzate; ribosomal	[11]
VVKL	antioxidative	marine Pisces	*Katsuwonus pelamis* Linnaeus	/	4	Whole protein hydrolyzate; ribosomal	[7]
VWIG	ACE inhibitor	marine Pisces	*Thunnus thynnus* Linnaeus	110	4	Whole protein hydrolyzate; ribosomal	[12]

YKYY	ACE inhibitor	marine algae	*Undaria pinnatifida* (Harvey) Suringar	64.2	4	Whole protein hydrolyzate; ribosomal	[8]
YKYY	ACE inhibitor	marine algae	*Undaria pinnatifida* (Harvey) Suringar	90.5	4	Whole protein hydrolyzate; ribosomal	[24]
YNKL	ACE inhibitor	marine algae	*Undaria pinnatifida* (Harvey) Suringar	64.2	4	Whole protein hydrolyzate; ribosomal	[24]
YRPY	ACE inhibitor	marine Pisces	*Pollachius pollachius* Linnaeus	320	4	Whole protein hydrolyzate; ribosomal	[8]
ALPHA	ACE inhibitor	marine Pisces	*Katsuwonus pelamis* Linnaeus	10	5	Whole protein hydrolyzate; ribosomal	[14]
ALSAF	antioxidative	marine Bivalvia	*Mactra quadrangularis* Reeve	/	5	Whole protein hydrolyzate; ribosomal	[1]
BELYP	ACE inhibitor	marine Cephalopoda	*Sepia officinalis* Linnaeus	5.22	5	Whole protein hydrolyzate; ribosomal	[21]
FDEQE	antioxidative	marine Bivalvia	*Mactra quadrangularis* Reeve	/	5	Whole protein hydrolyzate; ribosomal	[1]
FGHPY	antioxidative	marine Bivalvia	*Mytilus edulis* Linnaeus	/	5	Whole protein hydrolyzate; ribosomal	[27]
FQPSF	ACE inhibitor	marine Pisces	*Raja clavata* Linnaeus	12.56	5	Whole protein hydrolyzate; ribosomal	[27/28]
FVAGK	antioxidative	marine Pisces	*Katsuwonus pelamis* Linnaeus	/	5	Whole protein hydrolyzate; ribosomal	[7]

GAERP	antioxidative	marine Pisces	*Mustelus griseus* Pietschmann	/	5	Whole protein hydrolyzate; ribosomal	[20]
IITNW	ACE inhibitor	marine Pisces	*Raja clavata* Linnaeus	30.96	5	Whole protein hydrolyzate; ribosomal	[28]
IWHHT	ACE inhibitor	marine Pisces	*Katsuwonus pelamis* Linnaeus	5.8	5	Whole protein hydrolyzate; ribosomal	[14]
IYEGY	ACE inhibitor	marine Pisces	*Raja clavata* Linnaeus	79.42	5	Whole protein hydrolyzate; ribosomal	[28]
LKYPI	ACE inhibitor	marine Pisces	*Raja clavata* Linnaeus	27.07	5	Whole protein hydrolyzate; ribosomal	[28]
LYEGY	antioxidative	marine Bivalvia	*Mactra quadrangularis* Reeve	/	5	Whole protein hydrolyzate; ribosomal	[1]
MILMR	antioxidative	marine Pisces	*Larimichthys crocea* Richardson	/	5	Whole protein hydrolyzate; ribosomal	[29]
MMLDF	immunomodulating	marine algae	*Limnospira maxima* (Setchell & N.L.Gardner) Nowicka-Krawczyk, Mühlsteinová & Hauer	/	5	Whole protein hydrolyzate; ribosomal	[30]
MMLDF	ACE inhibitor	marine algae	*Limnospira maxima* (Setchell & N.L.Gardner) Nowicka-Krawczyk, Mühlsteinová & Hauer	/	5	Whole protein hydrolyzate; ribosomal	[30]

TEFTK	antioxidative	marine Bivalvia	*Mactra quadrangularis* Reeve	/	5	Whole protein hydrolyzate; ribosomal	[1]
TERGY	antioxidative	marine Bivalvia	*Mactra quadrangularis* Reeve	/	5	Whole protein hydrolyzate; ribosomal	[1]
TLKYP	ACE inhibitor	marine Pisces	*Raja clavata* Linnaeus	170	5	Whole protein hydrolyzate; ribosomal	[28]
VLYEE	antioxidative	marine Pisces	*Larimichthys crocea* Richardson	/	5	Whole protein hydrolyzate; ribosomal	[29]
VPSGK	antioxidative	marine Pisces	*Katsuwonus pelamis* Linnaeus	/	5	Whole protein hydrolyzate; ribosomal	[7]
VVPPA	ACE inhibitor	freshwater algae	*Chlorella vulgaris* Beijerinck	79.5	5	Whole protein hydrolyzate; ribosomal	[22]
YLMSR	antioxidative	marine Pisces	*Larimichthys crocea* Richardson	/	5	Whole protein hydrolyzate; ribosomal	[29]
DYGLYP	ACE inhibitor	marine Pisces	*Katsuwonus pelamis* Linnaeus	62	6	Whole protein hydrolyzate; ribosomal	[14]
ESAGIH	ACE inhibitor	marine Pisces	*Raja clavata* Linnaeus	371.6	6	Whole protein hydrolyzate; ribosomal	[28]
FHNMEK	antioxidative	marine Bivalvia	*Mactra quadrangularis* Reeve	/	6	Whole protein hydrolyzate; ribosomal	[1]
GALAAH	antioxidative	marine Pisces	*Sardinella aurita* Valenciennes	/	6	Whole protein hydrolyzate; ribosomal	[15]

GVYPHK	ACE inhibitor	marine Pisces	*Katsuwonus pelamis* Linnaeus	/	6	Whole protein hydrolyzate; ribosomal	[31]
HMPVTK	antioxidative	marine Bivalvia	*Mactra quadrangularis* Reeve	/	6	Whole protein hydrolyzate; ribosomal	[1]
IKPLNY	ACE inhibitor	marine Pisces	*Katsuwonus pelamis* Linnaeus	43	6	Whole protein hydrolyzate; ribosomal	[14]
IVGRPR	ACE inhibitor	marine Pisces	*Raja clavata* Linnaeus	1000	6	Whole protein hydrolyzate; ribosomal	[28]
KNGDGY	ACE inhibitor	marine Cephalopoda	*Sepia officinalis* Linnaeus	51.63	6	Whole protein hydrolyzate; ribosomal	[32]
LDAVNR	immunomodulating	marine algae	*Limnospira maxima* (Setchell & N.L.Gardner) Nowicka-Krawczyk, Mühlsteinová & Hauer	/	6	Whole protein hydrolyzate; ribosomal	[30]
LDAVNR	ACE inhibitor	marine algae	*Limnospira maxima* (Setchell & N.L.Gardner) Nowicka-Krawczyk, Mühlsteinová & Hauer	/	6	Whole protein hydrolyzate; ribosomal	[30]
LNGDVW	antioxidative	freshwater algae	*Chlorella ellipsoidea* Gerneck	/	6	Whole protein hydrolyzate; ribosomal	[11]
LPHSGY	antioxidative	marine Pisces	*Gadus chalcogrammus* Pallas	/	6	Whole protein hydrolyzate; ribosomal	[33]

PHHADS	antioxidative	marine Pisces	*Thunnus albacares* Bonnaterre	/	6	Whole protein hydrolyzate; ribosomal	[26]
PKAVHE	antioxidative	marine Pisces	*Thunnus albacares* Bonnaterre	/	6	Whole protein hydrolyzate; ribosomal	[26]
RSIKGF	ACE inhibitor	marine Cephalopoda	*Sepia officinalis* Linnaeus	32.74	6	Whole protein hydrolyzate; ribosomal	[32]
STHGVY	ACE inhibitor	marine Cephalopoda	*Sepia officinalis* Linnaeus	19.3	6	Whole protein hydrolyzate; ribosomal	[32]
WDDMEK	antioxidative	marine Bivalvia	*Mactra quadrangularis* Reeve	/	6	Whole protein hydrolyzate; ribosomal	[1]
YALPHA	ACE inhibitor	marine Cephalopoda	*Todarodes pacificus* Steenstrup	9.8	6	Whole protein hydrolyzate; ribosomal	[34]
ASFSPWG	immunomodulatory	marine Crustacea	*Penaeus vannamei* Boone	/	6	Whole protein hydrolyzate; ribosomal	[35]
DMIPAQK	ACE inhibitor	marine Pisces	*Katsuwonus pelamis* Linnaeus	45	7	Whole protein hydrolyzate; ribosomal	[14]
EKSYELP	ACE inhibitor	marine Cephalopoda	*Sepia officinalis* Linnaeus	14.41	7	Whole protein hydrolyzate; ribosomal	[32]
FEQDWAS	ACE inhibitor	marine algae	*Palmaria palmata* (Linnaeus) F.Weber & D.Mohr	2.8	7	Whole protein hydrolyzate; ribosomal	[16]
GIHETTY	ACE inhibitor	marine Cephalopoda	*Sepia officinalis* Linnaeus	25.66	7	Whole protein hydrolyzate; ribosomal	[32]

GMNNLTP	ACE inhibitor	marine algae	*Nannochloropsis oculata* (Droop) D.J.Hibberd	123	7	Whole protein hydrolyzate; ribosomal	[17]
GWNQWFL	antioxidative	marine algae	*Navicula salinicola* Hustedt	/	7	Whole protein hydrolyzate; ribosomal	[36]
GYALPHA	ACE inhibitor	marine Cephalopoda	*Todarodes pacificus* Steenstrup	27.3	7	Whole protein hydrolyzate; ribosomal	[34]
HFGDPFH	antioxidative	marine Bivalvia	*Mytilus edulis* Linnaeus	/	7	Whole protein hydrolyzate; ribosomal	[37]
HGPLGPL	antioxidative	marine Pisces	*Johnius belangerii* Cuvier	/	7	Whole protein hydrolyzate; ribosomal	[38]
LGPLGHQ	ACE inhibitor	marine Pisces	*Okamejei kenojei* Müller & Henle	4.22	7	Whole protein hydrolyzate; ribosomal	[39]
MAGVDHI	antidiabetic	marine algae	*Palmaria palmata* (Linnaeus) F.Weber & D.Mohr	159.37	7	Whole protein hydrolyzate; ribosomal	[23]
NGLEGLK	antioxidative	marine Cephalopoda	*Dosidicus gigas* d'Orbigny	/	7	Whole protein hydrolyzate; ribosomal	[37]
PMDYMVT	antioxidative	marine Pisces	*Thunnus tonggol* Bleeker	/	7	Whole protein hydrolyzate; ribosomal	[40]
PMDYMVT	immunomodulating	marine Pisces	*Thunnus tonggol* Bleeker	/	7	Whole protein hydrolyzate; ribosomal	[40]
SHDAHPE	antioxidative	marine Pisces	*Thunnus albacares* Bonnaterre	/	7	Whole protein hydrolyzate; ribosomal	[26]

VDHDHPE	antioxidative	marine Pisces	*Thunnus albacares* Bonnaterre	/	7	Whole protein hydrolyzate; ribosomal	[26]
VPAAPPK	ACE inhibitor	marine Pisces	*Channa striata* Bloch	0.45	7	Whole protein hydrolyzate; ribosomal	[41]
WPRGYFL	ACE inhibitor	freshwater algae	*Tetradesmus obliquus* (Turpin) M.J.Wynne	/	7	Whole protein hydrolyzate; ribosomal	[42]
WPRGYFL	antioxidative	freshwater algae	*Tetradesmus obliquus* (Turpin) M.J.Wynne	/	7	Whole protein hydrolyzate; ribosomal	[42]
AFVGYVLP	ACE inhibitor	marine Cephalopoda	*Sepia officinalis* Linnaeus	18.02	8	Whole protein hydrolyzate; ribosomal	[32]
ASFSPWGG	immunomodulatory	marine Crustacea	*Penaeus vannamei* Boone	/	8	Whole protein hydrolyzate; ribosomal	[35]
FGASTRGA	ACE inhibitor	marine Pisces	*Gadus chalcogrammus* Pallas	14.7	8	Whole protein hydrolyzate; ribosomal	[43]
GEREANVM	antioxidative	marine Pisces	*Mustelus griseus* Pietschmann	/	8	Whole protein hydrolyzate; ribosomal	[20]
LQPGQGQQ	antioxidative	marine Pisces	*Sardina pilchardus* Walbaum	/	8	Whole protein hydrolyzate; ribosomal	[44]
NGTWFEPP	ACE inhibitor	marine Pisces	*Channa striata* Bloch	0.63	8	Whole protein hydrolyzate; ribosomal	[41]
PSHDAHPE	antioxidative	marine Pisces	*Thunnus albacares* Bonnaterre	/	8	Whole protein hydrolyzate; ribosomal	[26]

PTHIKWGD	ACE inhibitor	marine Pisces	*Thunnus thynnus* Linnaeus	1.5	8	Whole protein hydrolyzate; ribosomal	[45], [46]
SFHPYFSY	ACE inhibitor	marine Cephalopoda	*Sepia officinalis* Linnaeus	82.71	8	Whole protein hydrolyzate; ribosomal	[32]
VEGKSPNV	antioxidative	marine Pisces	*Scorpaena notata* Rafinesque	/	8	Whole protein hydrolyzate; ribosomal	[3]
VEGKSPNV	ACE inhibitor	marine Pisces	*Scorpaena notata* Rafinesque	/	8	Whole protein hydrolyzate; ribosomal	[3]
WHNVSGSP	antioxidative	marine Bivalvia	*Mactra quadrangularis* Reeve	/	8	Whole protein hydrolyzate; ribosomal	[1]
YSEMPPGK	antioxidative	marine Bivalvia	*Mactra quadrangularis* Reeve	/	8	Whole protein hydrolyzate; ribosomal	[1]
YWVTSGPK	antioxidative	marine Bivalvia	*Mactra quadrangularis* Reeve	/	8	Whole protein hydrolyzate; ribosomal	[1]
APSGFLGMR	immunomodulatory	marine Crustacea	*Penaeus vannamei* Boone	/	9	Whole protein hydrolyzate; ribosomal	[35]
FDSGPAGVL	antioxidative	marine Cephalopoda	*Dosidicus gigas* d'Orbigny	/	9	Whole protein hydrolyzate; ribosomal	[47]
GQKSVPEVR	antioxidative	marine Pisces	*Scorpaena notata* Rafinesque	/	9	Whole protein hydrolyzate; ribosomal	[3]
GQKSVPEVR	ACE inhibitor	marine Pisces	*Scorpaena notata* Rafinesque	/	9	Whole protein hydrolyzate; ribosomal	[3]

IVGRPRHQG	ACE inhibitor	marine Pisces	*Katsuwonus pelamis* Linnaeus	2.4	9	Whole protein hydrolyzate; ribosomal	[14]
LGLNGDDVN	antioxidative	marine Pisces	*Conger myriaster* Brevoort	/	9	Whole protein hydrolyzate; ribosomal	[48]
LPTSEAAKY	antioxidative	marine Pisces	*Thunnus tonggol* Bleeker	/	9	Whole protein hydrolyzate; ribosomal	[40]
LPTSEAAKY	immunomodulating	marine Pisces	*Thunnus tonggol* Bleeker	/	9	Whole protein hydrolyzate; ribosomal	[40]
MIFPGGPQL	ACE inhibitor	marine Pisces	*Limanda aspera* Pallas	22.3	9	Whole protein hydrolyzate; ribosomal	[49]
MVGSAPGVL	ACE inhibitor	marine Pisces	*Okamejei kenojei* Müller & Henle	3.09	9	Whole protein hydrolyzate; ribosomal	[39]
PGPLGLTGP	ACE inhibitor	marine Pisces	*Okamejei kenojei* Müller & Henle	95	9	Whole protein hydrolyzate; ribosomal	[50]
VAMVPPFET	antioxidative	marine Bivalvia	*Mactra quadrangularis* Reeve	/	9	Whole protein hydrolyzate; ribosomal	[1]
VEVLPPAEL	antioxidative	marine algae	*Navicula salinicola* Hustedt	/	9	Whole protein hydrolyzate; ribosomal	[36]
APSGFLGMRG	immunomodulatory	marine Crustacea	*Penaeus vannamei* Boone	/	10	Whole protein hydrolyzate; ribosomal	[35]
DLGLGLPGAH	antioxidative	marine animal (Rotifera)	*Brachionus rotundiformis* Tschugunoff	/	10	Whole protein hydrolyzate; ribosomal	[51]

LLGPGLTNHA	antioxidative	marine animal (Rotifera)	*Brachionus rotundiformis* Tschugunoff	/	10	Whole protein hydrolyzate; ribosomal	[51]
PTAEGVYMVT	immunomodulating	marine Pisces	*Thunnus tonggol* Bleeker	8.8	10	Whole protein hydrolyzate; ribosomal	[52]
RPDFDLEPPY	antioxidative	marine Pisces	*Limanda aspera* Pallas	/	10	Whole protein hydrolyzate; ribosomal	[53]
WYGPDRPKFL	ACE inhibitor	freshwater algae	*Tetradesmus obliquus* (Turpin) M.J.Wynne	/	10	Whole protein hydrolyzate; ribosomal	[42]
WYGPDRPKFL	antioxidative	freshwater algae	*Tetradesmus obliquus* (Turpin) M.J.Wynne	/	10	Whole protein hydrolyzate; ribosomal	[42]
GPDRPKFLGPF	ACE inhibitor	freshwater algae	*Tetradesmus obliquus* (Turpin) M.J.Wynne	/	11	Whole protein hydrolyzate; ribosomal	[42]
GPDRPKFLGPF	antioxidative	freshwater algae	*Tetradesmus obliquus* (Turpin) M.J.Wynne	/	11	Whole protein hydrolyzate; ribosomal	[42]
LNLPTAVYMVT	antioxidative	marine Pisces	*Thunnus obesus* Lowe	/	11	Whole protein hydrolyzate; ribosomal	[54]
MIFPGAGGPEL	ACE inhibitor	marine Pisces	*Limanda aspera* Pallas	0.03	11	Whole protein hydrolyzate; ribosomal	[12]
VECYGPNRPQF	antioxidative	freshwater algae	*Chlorella vulgaris* Beijerinck	/	11	Whole protein hydrolyzate; ribosomal	[55]
VECYGPNRPQF	immunomodulating	freshwater algae	*Chlorella vulgaris* Beijerinck	/	11	Whole protein hydrolyzate; ribosomal	[55]

VECYGPNRPQF	antithrombotic	freshwater algae	*Chlorella vulgaris* Beijerinck	/	11	Whole protein hydrolyzate; ribosomal	[55]
VECYGPNRPQF	ACE inhibitor	freshwater algae	*Chlorella vulgaris* Beijerinck	29.6	11	Whole protein hydrolyzate; ribosomal	[55]
NGPLQAGQPGER	antioxidative	marine Cephalopoda	*Dosidicus gigas* d'Orbigny	/	12	Whole protein hydrolyzate; ribosomal	[47]
QQPHSRKGFPGP	antioxidative	marine Pisces	*Scorpaena notata* Rafinesque	/	12	Whole protein hydrolyzate; ribosomal	[3]
QQPHSRKGFPGP	ACE inhibitor	marine Pisces	*Scorpaena notata* Rafinesque	/	12	Whole protein hydrolyzate; ribosomal	[3]
SPGSSGPQGFTG	antidiabetic	marine Pisces	*Hippoglossus stenolepis* Schmidt	101.6	12	Whole protein hydrolyzate; ribosomal	[56]
CAYQWQRPVDRIR	antidiabetic	marine Pisces	*Thunnus albacares* Bonnaterre	78	13	Whole protein hydrolyzate; ribosomal	[57]
IPGDPGPPGPPGP	antidiabetic	marine Pisces	*Oreochromis niloticus* Linnaeus	65.4	13	Whole protein hydrolyzate; ribosomal	[56]
IRLIIVLMPILMA	ACE inhibitor	marine algae	*Palmaria palmata* (Linnaeus) F.Weber & D.Mohr	3300	13	Whole protein hydrolyzate; ribosomal	[58]
IRLIIVLMPILMA	antioxidative	marine algae	*Palmaria palmata* (Linnaeus) F.Weber & D.Mohr	/	13	Whole protein hydrolyzate; ribosomal	[58]
LKQELEDLLEKQE	antioxidative	marine Bivalvia	*Crassostrea gigas* Thunberg	/	13	Whole protein hydrolyzate; ribosomal	[59]

LPGERGRPGAPGP	antidiabetic	marine Pisces	*Oreochromis niloticus* Linnaeus	76.8	13	Whole protein hydrolyzate; ribosomal	[56]
NADFGLNGLEGLA	antioxidative	marine Cephalopoda	*Dosidicus gigas* d'Orbigny	/	13	Whole protein hydrolyzate; ribosomal	[60]
PACGGFWISGRPG	antidiabetic	marine Pisces	*Thunnus albacares* Bonnaterre	96.4	13	Whole protein hydrolyzate; ribosomal	[57]
VKAGFAWTANQQLS	antioxidative	marine Pisces	*Thunnus albacares* Bonnaterre	/	14	Whole protein hydrolyzate; ribosomal	[61]
GPLGLLGFLGPLGLS	antioxidative	marine Cephalopoda	*Dosidicus gigas* d'Orbigny	/	15	Whole protein hydrolyzate; ribosomal	[62]
GPLGLLGFLGPLGLS	ACE inhibitor	marine Cephalopoda	*Dosidicus gigas* d'Orbigny	90.03	15	Whole protein hydrolyzate; ribosomal	[62]
GPVGPAGNPGANGLN	antidiabetic	marine Pisces	*Hippoglossus stenolepis* Schmidt	81.3	15	Whole protein hydrolyzate; ribosomal	[56]
PGVGGPLGPIGPCYE	antidiabetic	marine Pisces	*Thunnus albacares* Bonnaterre	116.1	15	Whole protein hydrolyzate; ribosomal	[57]
RVAPEEHPVEGRYLV	immunomodulating	marine Bivalvia	*Cyclina sinensis* Gmelin	/	15	Whole protein hydrolyzate; ribosomal	[63]
TDGSEDYGILEIDSR	antithrombotic	marine Pisces	*Limanda aspera* Pallas	/	15	Whole protein hydrolyzate; ribosomal	[64]
ESTVPERTHPACPDFN	antioxidative	marine Pisces	*Johnius belangerii* Cuvier	/	16	Whole protein hydrolyzate; ribosomal	[65]

GPKGDRGLPGPPGRDG	antidiabetic	marine Pisces	*Oreochromis niloticus* Linnaeus	89.6	16	Whole protein hydrolyzate; ribosomal	[56]
PPGPTGPRGQPGNIGF	antidiabetic	marine Pisces	*Hippoglossus stenolepis* Schmidt	146.7	16	Whole protein hydrolyzate; ribosomal	[56]
KWCFRVCYRGICYRRCR	immunomodulating	marine animal (Xiphosurida)	*Limulus* O.F. Müller	/	17	Whole protein hydrolyzate; ribosomal	[66]
RWCFRVCYRGICYRRCR	immunomodulating	marine animal (Xiphosurida)	*Limulus* O.F. Müller	/	17	Whole protein hydrolyzate; ribosomal	[66]
EADIDGDGQVNYEEFVAMMTSK	antithrombotic	marine Bivalvia	*Mytilus edulis* Linnaeus	/	22	Whole protein hydrolyzate; ribosomal	[67]
GWGSFFKKAAHVGKHVGKAALTHYLG	antimicrobial	marine Pisces	*Pseudopleuronectes americanus* Walbaum	2.5	26	Whole protein hydrolyzate; ribosomal	[68]
GWGSFFKKAAHVGKHVGKAALTHYLG	immunomodulating	marine Pisces	*Pseudopleuronectes americanus* Walbaum	2.5	26	Whole protein hydrolyzate; ribosomal	[68]
RWRSFFKKAAHRGKHVGKRARTHYLG	antimicrobial	marine Pisces	*Pseudopleuronectes americanus* Walbaum	5	26	Whole protein hydrolyzate; ribosomal	[68]
RWRSFFKKAAHRGKHVGKRARTHYLG	immunomodulating	marine Pisces	*Pseudopleuronectes americanus* Walbaum	5	26	Whole protein hydrolyzate; ribosomal	[68]
SWSSFFKKAAHSGKHVGKSASTHYLG	antimicrobial	marine Pisces	*Pseudopleuronectes americanus* Walbaum	10	26	Whole protein hydrolyzate; ribosomal	[68]
SWSSFFKKAAHSGKHVGKSASTHYLG	immunomodulating	marine Pisces	*Pseudopleuronectes americanus* Walbaum	10	26	Whole protein hydrolyzate; ribosomal	[68]

SCASRCKSRCRARRCRYYVSVRYGGFCYCR C	antimicrobial	marine Bivalvia	*Mytilus edulis* Linnaeus	/	31	Whole protein hydrolyzate; ribosomal	[69]
GFFALIPKIISSPLFKTLLSAVGSALSSSGEQE	immunomodulatory	marine Pisces	*Pardachirus pavoninus* Lacepède	/	33	Whole protein hydrolyzate; ribosomal	[66]
GFFALIPKIISSPLFKTLLSAVGSALSSSGEQE	antimicrobial	marine Pisces	*Pardachirus pavoninus* Lacepède	/	33	Whole protein hydrolyzate; ribosomal	[66]
GCASRCKAKCAGRRCKGWASASFRGRCYCK CFRC	antimicrobial	marine Bivalvia	*Mytilus edulis* Linnaeus	/	34	Whole protein hydrolyzate; ribosomal	[69]
GCASRCKAKCAGRRCKGWASASFRRRCYCK CFRC	antimicrobial	marine Bivalvia	*Mytilus edulis* Linnaeus	/	34	Whole protein hydrolyzate; ribosomal	[69]
SCASRCKGHCRARRCGYYVSVLYRGRCYCK CLRC	antimicrobial	marine Bivalvia	*Mytilus edulis* Linnaeus	/	34	Whole protein hydrolyzate; ribosomal	[69]
GFGCPNNYACHQHCKSIRGYCGGYCAGWFR LRCTCYRCG	antimicrobial	marine Bivalvia	*Mytilus edulis* Linnaeus	/	39	Whole protein hydrolyzate; ribosomal	[69]
GFGCPNNYQCHRHCKSIPGRCGGYCGGWHR LRCTCYRGC	antimicrobial	marine Bivalvia	*Mytilus edulis* Linnaeus	/	39	Whole protein hydrolyzate; ribosomal	[69]
HPHVCTSYYCSKFCGTAGCTRYGCRNLHRG KLCFCLHCSR	antimicrobial	marine Bivalvia	*Mytilus edulis* Linnaeus	/	40	Whole protein hydrolyzate; ribosomal	[69]
HSHACTSYWCGKFCGTASCTHYLCRVLHPG KMCACVHCSR	antimicrobial	marine Bivalvia	*Mytilus edulis* Linnaeus	/	40	Whole protein hydrolyzate; ribosomal	[69]

*All taxonomical identifiers and authority have been verified for the most recent accepted form in the following databases: World Register of Marine Species [http://www.marinespecies.org]; AlgaeBase [www.algaebase.org]; FishBase [www.fishbase.se]. The reported species name might differ from the one reported in the original paper. Where the species name was not available a different taxonomical indicator has been employed.

Reference:

[1] Liu, R.; Zheng, W.; Li, J.; Wang, L.; Wu, H.; Wang, X.; Shi, L. Rapid identification of bioactive peptides with antioxidant activity from the enzymatic hydrolysate of *Mactra veneriformis* by UHPLC–Q-TOF mass spectrometry. *Food Chem.* **2015**, *167*, 484-489.

[2] Wu, H.; He, H.-L.; Chen, X.-L.; Sun, C.-Y.; Zhang, Y.- Z.; Zhou, B.-C. Purification and identification of novel angiotensin I-converting enzyme inhibitory peptides from shark meat hydrolysate. *Process Biochem.* **2008**, *43*, 457-461.

[3] Aissaoui, N.; Abidi, F.; Hardouin, J.; Abdelkafi, Z.; Marrakchi, N.; Jouenne, T.; Marzouki; N. ACE inhibitory and antioxidant activities of novel peptides from *Scorpaena notata* by-product protein hydrolysate. *Int. J. Pep. Res. Ther.* **2017**, 23, 13-23.

[4] Suetsuna, K.; Maekawa, K.; Chen, J. R. Antihypertensive effects *of Undaria pinnatifida* (wakame) peptide on blood pressure in spontaneously hypertensive rats. *J. Nutr. Biochem.* **2004**, *15(5)*, 267-272.

[5] Byun, H. G.; Kim, S. K. Structure and activity of angiotensin I-converting enzyme inhibitory peptides derived from Alaskan pollack skin. *Bioch. Mol. Biol.* **2002**, *35,* 239-243.

[6] Matsufuji, H.; Matsui, T.; Seki, E.; Osajima, K.; Nakashima, M.; Osajima, Y. Angiotensin I-converting enzyme inhibitory peptides in an alkaline proteinase hydrolysate derived from sardine muscle. *Biosci. Biotech. Biochem.* **1994**, *58*, 2244-2245.

[7] Suetsuna, K. Separation and identification of antioxidant peptides from proteolytic digest of dried bonito. *Nippon Suisan Gakkaishi (Japanese Edition)***1999**, *65*, 92-96.

[8] Meisel, H.; Walsh, D. J.; Murray, B.; FitzGerald, R. J. ACE inhibitory peptides. in*: Nutraceutical proteins and peptides in health and disease*. Mine, Y.; Shahidi, F. Eds. Publisher: CRC Taylor & Francis Group, Boca Raton, London, New York, **2006**, pp. 269-315.

[9] Ichimura, T.; Hu, J.; Aita, D. Q.; Maruyama, S. Angiotensin I-converting enzyme inhibitory activity and insulin secretion stimulative activity of fermented fish sauce. *J. Biosci. Bioeng.* **2003**, *96*, 496-499.

[10] Erdmann, K.; Grosser, N.; Schipporeit, K.; Schröder, H. The ACE inhibitory dipeptide Met-Tyr diminishes free radical formation in human endothelial cells via induction of heme oxygenase-1 and ferritin. *J. Nutr*. **2006**, *136*, 2148-2152.

[11] Ko, S. C.; Kang, N.; Kim E. A.; Kang, M. C.; Lee, S. H.; Kang, S. M.; Lee, J. B.; Jeon, B. T.; Kim, S. K.; Park, S. J.; Park, P. J.; Jung, W. K.; Kim, D.; Jeon, Y. J. A novel angiotensin I-converting enzyme (ACE) inhibitory peptide from a marine *Chlorella ellipsoidea* and its antihypertensive effect in spontaneously hypertensive rats. *Process Biochem.* **2012**, *47(12)*, 2005-2011.

[12] Murray, B. A.; FitzGerald, R. J. Angiotensin converting enzyme inhibitory peptides derived from food proteins: biochemistry, bioactivity and production. *Curr. Pharm. Des.* **2007**, *13*, 773-791.

[13] Suetsuna, K. Antioxidant peptides from the protease digest of prawn (*Penaeus japonicus*) muscle. *Mar. Biotechnol*. **2000**, *2*, 5-10.

[14] Yokoyama, K.; Chiba, H.; Yoshikawa, M. Peptide inhibitors for angiotensin I-converting enzyme from thermolysin digest of dried bonito. *Biosci. Biotech. Biochem*. **1992**, *56*, 1541-1545.

[15] Bougatef, A.; Nedjar-Arroume, N.; Manni, L.; Ravallec, R.; Barkia, A.; Guillochon, D.; Nasri, M. Purification and identification of novel antioxidant peptides from enzymatic hydrolysates of sardinelle (*Sardinella aurita*) by-products proteins. *Food Chem.* **2010**, *118*, 559-565.

[16] Furuta, T.; Miyabe, Y.; Yasui, H.; Kinoshita, Y.; Kishimura, H. Angiotensin I converting enzyme inhibitory peptides derived from phycobiliproteins of Dulse *Palmaria palmata. Mar. Drugs* **2016**, *14*, 32-42.

[17] Samarakoon, K. W.; O-Nam, K.; Ko, J. Y.; Lee, J. H.; Kang, M. C.; Kim, D.; Baek, J. L; Jung-Suck, L.; Jeon, Y. J. Purification and identification of novel angiotensin-I converting enzyme (ACE) inhibitory peptides from cultured marine microalgae (*Nannochloropsis oculata*) protein hydrolysate**.** *J. Appl. Phycol*. **2013**, *25*, 1595-1606.
[18] Ukeda, H.; Matsuda, H.; Osajima, K.; Matsufuji, H.; Matsui, T.; Osajima, Y. Peptides from peptic hydrolyzate of heated sardine meat that inhibit angiotensin I converting enzyme. *Nippon Nogeikagaku Kaishi* **1992**, *66*, 25-29.
[19] Liu, P.; Lan, X.; Yaseen, M.; Wu, S.; Feng, X.; Zhou, L.; Sun, J.; Liao, A.; Liao, D.; Sun, L. Purification, characterization and evaluation of inhibitory mechanism of ACE inhibitory peptides from pearl oyster (*Pinctada fucata martensii*) meat protein hydrolysate. *Mar. Drugs* **2019**, *17(8)*, E463.
[20] Tao, J.; Zhao, Y. Q.; Chi, C. F.; Wang, B. Bioactive peptides from cartilage protein hydrolysate of spotless smoothhound and their antioxidant activity *in vitro*. *Mar. Drugs* **2018**, *16(4),* pii: E100.
[21] Balti, R.; Nedjar-Arroume, N.; Adjé, E. Y.; Guillochon, D.; Nasri, M. Analysis of novel angiotensin I-converting enzyme inhibitory peptides from enzymatic hydrolysates of cuttlefish (*Sepia officinalis*) muscle proteins. *J. Agric. Food Chem.* **2010**, *58*, 3840-3846.
[22] Suetsuna, K.; Chen, J.-R. Identification of antihypertensive peptides from peptic digest of two microalgae, *Chlorella vulgaris* and *Spirulina platensis*. *Mar. Biotechnol.* **2001**, *3*, 305–309.
[23] Harnedy, P. A.; O'Keeffe, M. B.; FitzGerald, R. J. Purification and identification of dipeptidyl peptidase (DPP) IV inhibitory peptides from the macroalga *Palmaria palmata*. *Food Chem.* **2015**, *172*, 400-406.
[24] Suetsuna, K.; Nakano, T. Identification of an antihypertensive peptide from peptic digest of Wakame (*Undaria pinnatifida*). *J. Nutr. Biochem.* **2000**, *11(9)*, 450-454.
[25] Nguyen, M.; Zhong-Ji, Q.; Nguyen, V. T.; Choi, I. W.; Heo, S. J.; Oh, C.; Kang, D. H.; Kim, G.; Jung, W. K. Tetrameric peptide purified from hydrolysates of biodiesel byproducts of *Nannochloropsis oculata* induces osteoblastic differentiation through MAPK and Smad pathway on MG-63 and D1 cells. *Process Biochem.* **2013**, *48*, 1387-1394.
[26] Jao, C. L.; Ko, W. C. 1,1-Diphenyl-2-picrylhydrazyl (DPPH) radical scavenging by protein hydrolyzates from tuna cooking juice. *Fisheries Sci.* **2002**, *68*, 430-435.
[27] Jung, W. K.; Rajapakse, N.; Kim, S. K. Antioxidative activity of a low molecular weight peptide derived from the sauce of fermented blue mussel, *Mytilus edulis*. *Eur. Food Res. Technol.* **2005**, *220*, 535-539.
[28] Lassoued, I.; Mora, L.; Barkia, A. M. C.; Nasri, M.; Toldra, F. Angiotensin I-converting enzyme inhibitory peptides FQPSF and LKYPI identified in *Bacillus subtilis* A26 hydrolysate of thornback ray muscle. *Int. J. Food Sci. Food Technol.* **2016**, *51*, 1604-1609.
[29] Chi, C. F.; Hu F. Y.; Wang, B.; Ren, X. J.; Deng, S. G.; Wu, C. W. Purification and characterization of three antioxidant peptides from protein hydrolyzate of croceine croaker (*Pseudosciaena crocea*) muscle. *Food Chem.* **2015**, *168,* 662-667.
[30] Vo, T. S.; Ryu, B.; Kim, S. K. Purification of novel anti-inflammatory peptides from enzymatic hydrolysate of the edible microalgal *Spirulina maxima*. *J. Funct. Foods* **2013**, *5*,1336–1346.
[31] Karaki, H.; Kuwahara, M.; Sugano, S.; Doi, C.M.; Doi, K.; Matsumura, N.; Shimizu, T. Oral administration of peptides derived from bonito bowels decreases blood pressure in spontaneously hypertensive rats. *Comp. Biochem. Physiol.* **1993**, *104C*, 351-353.

[32] Balti, R.; Bougatef, A.; Sila, A.; Guillochon, D.; Dhulster, P.; Nedjar-Arroume, N. Nine novel angiotensin I-converting enzyme (ACE) inhibitory peptides from cuttlefish (*Sepia officinalis*) muscle protein hydrolysates and antihypertensive effect of the potent active peptide in spontaneously hypertensive rats. *Food Chem.* **2015**, *170*, 519-525.
[33] Je, J.-Y.; Park, P.-J.; Kim, S.-K. Antioxidant activity of a peptide isolated from Alaska pollack (*Theragra chalcogramma*) frame protein hydrolysate. *Food Res. Int.* **2005**, 38, 45–50.
[34] Wako, Y.; Ishikawa, S.; Muramoto, K. Angiotensin I-converting enzyme inhibitors in autolysates of squid liver and mantle muscle. *Biosci. Biotech. Biochem.* **1996**, *60*, 1353-1355.
[35] Nieto, J.; Veelaert, D.; Derua, R.; Waelkens, E.; Cerstiaens, A.; Coast, G.; Devreese, B.; Van Beeumen, J.; Calderon, J.; De Loof, A.; Schoofs, L. Identification of one tachykinin and two kinin related peptides in the brain of the white shrimp, *Penaeus vannamei*. *Biochem. Biophys. Res. Comm.* **1998**, *248*, 406-411.
[36] Kang, K. H.; Qian, Z. J.; Ryu, B.; Karadeniz, F.; Kim, D.; Kim, S. K. Antioxidant peptides from protein hydrolysate of microalgae *Navicula incerta* and their protective effects in HepG2/CYP2E1 cells induced by ethanol. *Phytother Res.* **2012**, *26(10)*, 1555-1563. doi: 10.1002/ptr.460
[37] Rajapakse, N.; Mendis, E.; Jung, W.-K.; Je, J.-Y.; Kim, S.-K. Purification of a radical scavenging peptide from fermented mussel sauce and its antioxidant properties. *Food Res. Int.* **2005**, *38*, 175-182.
[38] Mendis, E.; Rajapakse, N.; Kim, S.-K. Antioxidant properties of a radical-scavenging peptide purified from enzymatically prepared fish skin gelatin hydrolysate. *J. Agric. Food Chem.* **2005**, *53*, 581–587.
[39] Ngo, D. H.; Ryu, B.; Kim, S. K. Active peptides from skate (*Okamejei kenojei*) skin gelatin diminish angiotensin-I converting enzyme activity and intracellular free radical-mediated oxidation. *Food Chem.* **2014**, *143*, 246-255.
[40] Hsu, K.-C. Purification of antioxidative peptides prepared from enzymatic hydrolysates of tuna dark muscle by-products. *Food Chem.* **2010**, *122*, 42-48.
[41] Ghassem, M.; Arihara, K.; Babji, A. S.; Said, M.; Ibrahim, S. Purification and identification of ACE inhibitory peptides from Haruan (*Channa striatus*) myofibrillar protein hydrolysate using HPLC–ESI-TOF MS/MS. *Food Chem.* **2011**, *129*, 1770-1777. doi:10.1016/j.foodchem.2011.06.051
[42] Montone, C. M.; Capriotti, A. L.; Cavaliere, C.; La Barbera, G.; Piovesana, S.; Zenezini Chiozzi, R.; Lagana', A. Peptidomic strategy for purification and identification of potential ACE-inhibitory and antioxidant peptides in *Tetradesmus obliquus* microalgae. *Anal. Bioanal. Chem.* **2018**, *410*, 3573-3586.
[43] Je, J.-Y.; Park, P.-J.; Kwon, J. Y.; Kim, S.-K. A novel angiotensin I converting enzyme inhibitory peptide from Alaska Pollack (*Theragra chalcogramma*) frame protein hydrolysate. *J. Agric. Food. Chem.* **2004**, *52*, 7842–7845.
[44] Suetsuna, K.; Ukeda, H. Isolation of an octapeptide which possesses active oxygen scavenging activity from peptic digest of sardine muscle. *Nippon Suisan Gakkaishi* (Japanese Edition) **1999**, *65*, 1096-1099.
[45] Kohama, Y.; Matsumoto, S.; Oka, H.; Teramoto, T.; Okabe, M.; Mimura, T. Isolation of angiotensin-converting enzyme inhibitor from tuna muscle. *Biochem. Biophys. Res. Commun.* **1988**, *155*, 332-337,
[46] Hasan, F.; Kitagawa, M.; Kumada, Y.; Hashimoto, N.; Shiiba, M.; Katoh, S.; Terashima, M. Production kinetics of angiotensin-I converting enzyme inhibitory peptides from bonito meat in artificial gastric juice. *Process Biochem.* **2006**, *41*, 505-511.

[47] Mendis, E.; Rajapakse, N.; Byun, H.-G.; Kim, S.-K. Investigation of jumbo squid (*Dosidicus gigas*) skin gelatin peptides for their in vitro antioxidant effects. *Life Sci.* **2005**, *77*, 2166-2178.
[48] Ranathunga, S.; Rajapakse, N.; Kim, S.-K. Purification and characterization of antioxidative peptide derived from muscle of conger eel (*Conger myriaster*). *Eur. Food Res. Technol.* **2006**, *222*, 310-315.
[49] He, H. L.; Liu, D.; Ma, C. B. Review on the angiotensin-I-converting enzyme (ACE) inhibitor peptides from marine proteins. *Appl. Biochem. Biotechnol.* **2013**, *169(3),* 738-749.
[50] Lee, J. K.; Jeon, J.-K.; Byun, H.-G. Effect of angiotensin-converting enzyme inhibitory peptide purified from skate skin hydrolysate. *Food Chem.* **2011**, *125*, 495-499.
[51] Byun, H.-G.; Lee, J. K.; Park, H. G.; Jeon, J.-K.; Kim, S.-K. Antioxidant peptides isolated from the marine rotifer (*Brachionus rotundiformis*). *Process Biochem.* **2009**, *44*, 842-846.
[52] Hsu, K.-C.; Li-Chan, E. C. Y.; Jao, C.-L. Antiproliferative activity of peptides prepared from enzymatic hydrolysates of tuna dark muscle on human breast cancer cell line MCF-7. *Food Chem.* **2011**, *126*, 517-622.
[53] Jun, S.-Y.; Park, P.-J.; Jung, W.-K.; Kim, S.-K. Purification and characterization of an antioxidative peptide from enzymatic hydrolysate of yellowfin sole (*Limanda aspera*) frame protein. *Eur. Food Res. Technol.* **2004**, *219*, 20-26.
[54] Je, J.-Y.; Qian, Z.-J.; Lee, S.-H.; Byun, H.-G.; Kim, S.-K. Purification and antioxidant properties of bigeye tuna (*Thunnus obesus*) dark muscle peptide on free radical-mediated oxidative systems. *J. Med. Food* **2008**, *11*, 629-637.
[55] Sheih, I.-C.; Fang, T. J.; Wu, T.-K.; Lin, P.-H. Anticancer and antioxidant activities of the peptide fraction from algae protein waste. *J. Agric. Food Chem.* **2010**, *58*, 1202-1206.
[56] Wang, T. Y.; Hsieh, C. H.; Hung, C. C.; Jao, C. L.; Chen, M. C.; Hsu, K. C. Fish skin gelatin hydrolysates as dipeptidyl peptidase IV inhibitors and glucagon-like peptide-1 stimulators improve glycaemic control in diabetic rats: A comparison between warm- and cold-water fish. *J. Funct. Foods* **2015**, *18*, 330–340.
[57] Huang, S.-L.; Jao, C.-L.; Hoc, K.-P.; Hsu, K.-C. Dipeptidyl-peptidase IV inhibitory activity of peptides derived from tuna cooking juice hydrolysates. *Peptides* **2012**, *35*, 114-121.
[58] Fitzgerald, C.; Mora-Soler, L.; Gallagher, E.; O'Connor, P.; Prieto, J.; Soler-Vila, A.; Hayes, M. Isolation and characterization of bioactive pro-peptides with in vitro renin inhibitory activities from the macroalga *Palmaria palmata*. *J. Agric. Food Chem.* **2012**, *60*, 7421–7427.
[59] Qian, Z.-J.; Jung, W.-K.; Byun, H.-G.; Kim, S.-K. Protective effect of an antioxidative peptide purified from gastrointestinal digests of oyster, *Crassostrea gigas* against free radical induced DNA damage. *Bioresour. Technol.* **2008**, *99*, 3365-3371.
[60] Rajapakse, N.; Mendis, E.; Byun, H.-G.; Kim, S.-K. Purification and in vitro antioxidative effects of giant squid muscle peptides on free radical-mediated oxidative systems. *J. Nutr. Biochem.* **2005**, *16*, 562-569.
[61] Je, J.-Y.; Qian, Z.-J.; Byun, H.-G.; Kim, S.-K. Purification and characterization of an antioxidant peptide obtained from tuna backbone protein by enzymatic hydrolysis. *Process Biochem.* **2007**, *42*, 840-846.
[62] Alemán, A.; Giménez, B.; Pérez-Santin, E.; Gómez-Guillén, M. C.; Montero, P. Contribution of Leu and Hyp residues to antioxidant and ACE-inhibitory activities of peptide isolated from squid gelatin hydrolysate. *Food Chem.* **2011**, *125*, 334-341.

[63] Li, W.; Ye, S.; Zhang, Z.; Tang, J.; Jin, H.; Huang, F.; Yang, Z.; Tang, Y.; Chen, Y.; Ding, G.; et al. Purification and characterization of a novel pentadecapeptide from protein hydrolysates of *Cyclina sinensis* and its immunomodulatory effects on RAW264.7 cells. *Mar. Drugs* **2019**, *17*, E30.
[64] Rajapakse, N.; Jung, W.-K.; Mendis, E.; Moon, S.-H.; Kim, S.-K. A novel anticoagulant purified from fish protein hydrolysate inhibits factor XIIa and platelet aggregation. *Life Sci.* **2005**, *76*, 2607-2619.
[65] Kim, S.-Y.; Je, J.-Y.; Kim, S.-K. Purification and characterization of antioxidant peptide from hoki (*Johnius belengerii*) frame protein by gastrointestinal digestion. *J. Nutr. Biochem.* **2007**, *18*, 31-38.
[66] Saberwal, G.; Nagaraj, R. Cell-lytic and antibacterial peptides that act by perturbing the barrier function of membranes: facets of their conformational features, structure-function correlations and membrane-perturbing abilities. *Biochim. Biophys. Acta* **1994**, *1197*, 109-131.
[67] Jung, W. K; Kim, S.-K. Isolation and characterization of an anticoagulant oligopeptide from blue mussel, *Mytilus edulis*. *Food Chem.* **2009**, *117*, 687-692.
[68] Sung, W. S.; Lee, D. G. Pleurocidin-derived antifungal peptides with selective membrane-disruption effect. *Biochem. Biophys. Res. Commun.*, **2008**, *369*, 858-861.
[69] Mitta, G.; Vandelbulcke, N. T.; Romestand, B.; Beauvillain, S. M.; Roch, P. Differential distribution and defence involvement of antimicrobial peptides in mussel. *J. Cell Sci.* **2000**, *113*, 2759-2769.

Table S2: Brief overview of principal mechanisms behind different bioactivities and quick comparison between different bioassays used to assess these activities, with related references

Bioactivities	Main mechanism(s)	Brief comparison of bioassays	References
ACE inhibitors	Both competitive and noncompetitive inhibitors of angiotensin-converting enzyme (ACE).	*In vitro* measurement of ACE-inhibition: quick, relatively cheap, but sensitivity is affected by the type of compound used as a substrate (since type of substrate would dictate method of product detection).<br>*In vivo* detections of changes in systolic and diastolic blood pressures: slow and highly dependent of age and hormonal status.	[1-8]
antioxidative	Scavenging of free radicals, prevention of lipid peroxidation and/or metal chelation.	Testing is usually done *in vitro*, but direct comparison between different tests' specificity and sensitivity is hampered by three factors: 1. Different tests are measuring different parameters (for example, certain tests, such as oxygen radical absorbance detect transfer of hydrogen ions, others like DPPH (1,1-diphenyl-2-picrylhydrazyl) and ABTS (2,2'-azino-bis-3ethylbenzthiazoline-6-sulfonic acid) measure transfer of electrons, β-carotene bleaching assay determine rate of lipid oxidation, etc.); 2. Due to the usage of different substrates, results of certain assays (especially DPPH and ABTS) could be expressed in different units; 3. Sensitivity of tests that measure electron transfer is also depended on polarity (for example, DPPH shows higher sensitivity and reproducibility when hydrophilic substrates are detected).	[9-16]
immunomodulating	The highest number of possible mechanisms – changes in gene expression (especially genes coding for caspases and genes coding for proteins involved in signaling transduction pathways such as MAPK and NF-kB), inhibition of cell proliferation (often through microtubule inhibition), inhibition of protein synthesis (sometimes through direct interaction with ribosome), antinflammatory effect through inhibition of monocytes binding to endothelia, regulation of cytokine levels (especially TNF), cytotoxic effect through	Both *in vitro* and *in vivo* tests are used for assessing immunomodulatory effect. In both *in vivo* and *in vitro* testing different activities are assessed with different assays (for example, cytotoxic activity *in vitro* can be assessed using staining tests, such as acid phosphatase assay, neutral red uptake assay, lactate dehydrogenase assay, etc; apoptosis can be measured by caspase assay and monitoring changes in mitochondrial transmembrane potential or DNA synthesis); cytokine release in vitro is often monitored by ELISA (enzyme-linked immunosorbant assay), etc.; *in vivo* experiments include monitoring in weight changes, changes in cytokine profiles, occurrence of swelling, measuring indexes of spleen and thymus status, monitoring hemolysin content and release of histamine, etc. Similar as with monitoring antihypertensive effect *in vivo*, immunological effect *in vivo* depends of variety of factors (age, sex, nutrition, etc.). Therefore, reproducibility of results is low.	[17-24]

	opening of voltage-gated membrane channels, etc.	Additionally, since *in vivo* experiments are done in rats, comparison of rat and human immune response is debatable.	
antidiabetic	Usually competitive inhibitors of dipeptidyl peptidase IV (DPPIV); however interaction with glucagon-like peptide (GLP) receptor and glucose-dependent insulinotropic polypeptide (*GIP*) were also reported, as well as changes in gene expression (in particular up-regulation of genes encoding glucose transporters –GLUT4).	*In vitro* tests usually include measurement of enzymatic activities of enzyme such as dipeptidyl peptidase IV, α-glucosidase, α-amylase, N-acetyl-glucosaminidase, etc. Percentage of inhibition of these enzymes is usually determined using spectrometry (due to the usage of colored substrates and forming of colored products). These methods are fast, relatively inexpensive and have relatively high reproducibility.<br>*In vivo* assays either determine concentration of glucose in blood (with HPLC (high performance liquid chromatography) being method of choice, due to its high sensitivity and reproducibility) or formation of AGE (advanced glycosylated end products). Recently, technique that employs gold labeled monoclonal and polyclonal antibodies against AGE products is becoming popular due to low detection limit, specificity and high reproducibility of results.	[25-32]
antimicrobial	Several mechanisms are employed (often simultaneously): binding to specific compounds in bacterial membrane (for example, callinectin binds to anti-callinectin-like peptides) which results in formation of pores and cell lysis; phagocytosis as result of binding to compounds in cell wall (as in case of certain mytilins), lysis as result of changes in bacterial membrane potential (by, for example, blocking K^+ channels), inhibition of basic metabolic pathways (such as inhibition of protein and nucleic acid synthesis and oxidative phosphorylation) usually by binding to proteins crucial for these processes, inhibition of cell growth through direct targeting of bacterial membrane molecules (for example, lectins inhibit growth by binding to	Testing for antimicrobial activity is usually done *in vitro*. Two main categories of antimicrobial tests are assays measuring cell growth (like agar diffusion (inhibition zone) assay) and assays measuring cell viability (like MTT test, apoptosis assay, etc.). Inhibition zone assay is cheap, easy to perform, but gives no data about minimum inhibitory concentration (MIC) that serves as principal parameter of antimicrobial activity. This problem can be solved by so-called agar dilution methods where various concentrations of compound are incorporated into agar, allowing for calculation of MIC. MTT test assesses cell viability by measuring activity of NAD(P)H-dependent oxidoreductase enzymes. It is characterized by high sensitivity, but it is more costly and harder to perform compared to agar dilution test. Novel methods like qPCR, flow cytometry, fluorescence-based assays, etc. have good reproducibility and high sensitivity, but their high price limits their usage.	[33-43]

	mannans), inhibition of cell-cell signaling and formation of biofilms, activation of immune response (usually through activation of proinflammatory response).		
antithrombotic	Inhibition of platelet aggregation, due to inhibition of coagulation factors. This inhibition can be achieved either through formation of complexes (this is particular true for interactions with thrombin) or through enzymatic action (peptides acting like proteases and cleaving specific clotting factors in cascade). Additionally, peptides can cause depletion of Ca^{2+} ions from blood (thus interfering with signaling cascade).	Most commonly used *in vitro* assay is so-called APTT (activated partial thromboplastin time) assay that measures time necessary for a blood clot to form. Although this test is quick and easy to perform there are several issues that could affect reliability of results. First, quality of reagents and instrumentation can affect both sensitivity and reproducibility of results. Second, there is a number of interfering compounds (such as cholesterol, vitamin C, etc.) that falsely prolong clotting time. During *in vivo* tests, percentage of thrombosis inhibition is determined by firstly inducing lesions in endothelium (usually by using lasers) and then intravenously introducing bioactive peptide. Like previously stated, main problems with such *in vivo* tests are great variability between subjects, as well as difficulty of correlating results obtained with animal models with those obtained for humans.	[44-51]

[1] He, H.; Liu, D.; Ma, C. Review on the angiotensin-I-converting enzyme (ACE) inhibitor peptides from marine proteins. *Appl. Biochem. Biotechnol.* **2013**, *169*, 738–749. https://doi.org/10.1007/s12010-012-0024-y

[2] Abdelhedi, O.; Nasri, M. Basic and recent advances in marine antihypertensive peptides: Production, structure-activity relationship and bioavailability. *Trends Food Sci. Technol.* **2019**, *88*, 543-557. https://doi.org/10.1016/j.tifs.2019.04.002

[3] Pujiastuti, D.Y.; Amin, M.N.G.; Alamsjah, M.A.; Hsu, J.-L. Marine organisms as potential sources of bioactive peptides that inhibit the activity of angiotensin I-converting enzyme: a review. *Molecules* **2019**, *24(14)*, 2541. https://doi.org/10.3390/molecules24142541

[4] Abachi, S.; Bazinet, L.; Beaulieu, L. Antihypertensive and angiotensin-I-converting enzyme (ACE)-inhibitory peptides from fish as potential cardioprotective compounds. *Mar. Drugs* **2019**, *17*, 613. https://doi.org/10.3390/md17110613

[5] Qian, Z.J.; Je, J.Y.; Kim, S.K. Antihypertensive effect of angiotensin-I converting enzyme-inhibitory peptide from hydrolysates of bigeye tuna dark muscle, *Thunnus obesus*. *J. Agric. Food Chem*. **2007**, *55*, 8398–8403. https://doi.org/10.1021/jf501368a

[6] Duan, X.; Wu, F.; Li, M.; Yang, N.; Wu, C.; Jin, Y.; Xu, X. Naturally occurring angiotensin I-converting enzyme inhibitory peptide from a fertilized egg and its inhibitory mechanism. *J. Agric. Food Chem*. **2014**, *62(24)*, 5500–5506. https://doi.org/10.1021/jf0710635

[7] Balti, R.; Bougatef, A.; Sila, A.; Guillochon, D.; Dhulster, P.; Nedjar-Arroume, N. Nine novel angiotensin I-converting enzyme (ACE) inhibitory peptides from cuttlefish (*Sepia officinalis*) muscle protein hydrolysates and antihypertensive effect of the potent active peptide in spontaneously hypertensive rats. *Food Chem*. **2015**, *170*, 519–525. http://doi.org/10.1016/j.foodchem.2013.03.091

[8] Lee, S.-H.; Qian, Z.; Kim, S.-K. A novel angiotensin I converting enzyme inhibitory peptide from tuna frame protein hydrolysate and its antihypertensive effect in spontaneously hypertensive rats. *Food Chem.* **2010**, *118(1)*, 96–102. https://doi.org/10.1016/j.foodchem.2009.04.086

[9] Aissaoui, N.; Abidi, F.; Hardouin, J. ACE inhibitory and antioxidant activities of novel peptides from *Scorpaena notata* by-product protein hydrolysate. *Int. J. Pept. Res. Ther.* **2017**, *23*, 13-23. https://doi.org/10.1007/s10989-016-9536-6

[10] Ngo, D-H.; Vo, T.-S.; Ngo, D.-N.; Wijesekara, I.; Kim, S.-K. Biological activities and potential health benefits of bioactive peptides derived from marine organisms. *Int. J. Biol. Macromol.* **2012**, *51*, 378–383. http://dx.doi.org/10.1016/j.ijbiomac.2012.06.001

[11] Ngo, D-H.; Kim, S.-K. Marine bioactive peptides as potential antioxidants. *Curr. Protein Pep. Sci.* **2013**, *14(3)*, 189-198. http://doi.org/10.2174/13892037113149990041

[12] Wu, R.; Chen, L.;Liu, D; Huang, J; Zhang, J; Xiao, X; Lei, M; Chen, Y; He, H. Preparation of antioxidant peptides from salmon byproducts with bacterial extracellular proteases. *Mar. Drugs* **2017**, *15(1),* 4 -23. https://doi.org/10.3390/md15010004

[13] Tao, J.; Zhao, Y.Q.; Chi, C.F.; Wang, B. Bioactive peptides from cartilage protein hydrolysate of spotless smoothhound and their antioxidant activity *in vitro*. *Mar. Drugs* **2018**, *16(4)*, 100-117. https://doi.org/10.3390/md16040100

[14] Thaipong, K.; Boonprakob, U.; Crosby, K.; Cisneros-Zevallos, L.; Hawkins Byrne, D. Comparison of ABTS, DPPH, FRAP, and ORAC assays for estimating antioxidant activity from guava fruit extracts *J. Food Compost. Anal.* **2006**, *19*, 669-675. https://doi.org/10.1016/j.jfca.2006.01.003

[15] Floegel, A.; Kim, D.-O.; Chung, S.–J.; Koo, S.I.; Chun, O.K. Comparison of ABTS/DPPH assays to measure antioxidant capacity in popular antioxidant-rich US foods. *J. Food Compost. Anal.* **2011**, *24*, 1043-1048. https://doi.org/10.1016/j.jfca.2011.01.008

[16] Aloğulu, H.Ş.; Öner, Z. Determination of antioxidant activity of bioactive peptide fractions obtained from yogurt. *J. Dairy Sci.* **2011**, *94*, 5305-5314. https://doi.org/10.3168/jds.2011-4285

[17] Wang, Y.-K.; He, H.-L.; Wang, G.-F.; Wu, H.; Zhou, B.-C.; Chen, X.-L.; Zhang, Y.-Z. Oyster (*Crassostrea gigas*) hydrolysates produced on a plant scale have antitumor activity and immunostimulating effects in BALB/c mice. *Mar. Drugs* **2010**, *8*, 255-268; https://doi.org/10.3390/md8020255

[18] Subhan, F.; Kang, H. Y.; Lim, Y.; Ikram, M.; Baek, S.-Y.; Jin, S.; Jeong, Y. H.; Kwak, J. Y.; Yoon, S. Fish scale collagen peptides protect against CoCl2/TNF-*α* induced cytotoxicity and inflammation via inhibition of ROS, MAPK, and NF-κB pathways in HaCaT cells. *Oxid. Med. Cell Longev.* **2017**, Article ID 9703609. https://doi.org/10.1155/2017/9703609

[19] Agrawal, S.; Acharya, D.; Adholeya, A.; . Barrow, C. J.; Deshmukh, S. K. Nonribosomal peptides from marine microbes and their antimicrobial and anticancer potential. *Front. Pharmacol.* **2017**, *8*, 828. https://doi.org/10.3389/fphar.2017.00828

[20] Fan, M.; Nath, A. K.; Tang, Y.; Choi, Y.-J.; Debnath, T.; Choi, E.-J.; Kim, E.-K. Investigation of the anti-prostate cancer properties of marine-derived compounds. *Mar. Drugs* **2018**, *16*, 160; https://doi.org/10.3390/md16050160

[21] Romero, A.; Estevez-Calvar, N.; Dios, S.; Figueras, A.; Novoa, B. New insights into the apoptotic process in mollusks: characterization of caspase genes in *Mytilus galloprovincialis*. *PLoS ONE* **2011**, *6(2):* e17003. https://doi.org/10.1371/journal.pone.0017003

[22] Clay, T.M.; Hobeika, A.C.; Mosca, P.J.; Lyerly, H.K.; Morse, M. Assays for monitoring cellular immune responses to active immunotherapy of cancer. *Clin. Cancer Res*. **2011**, *7(5),* 1127–1135.
[23] Galli, S.J.; Borregaard, N.; Wynn, T.A. Phenotypic and functional plasticity of cells of innate immunity: Macrophages, mast cells and neutrophils. *Nat. Immunol*. **2011**, *12(11),* 1035–1044. https://doi.org/10.1038/ni.2109
[24] Maestri, E.; Marmiroli, M.; Marmiroli, N. Bioactive peptides in plant-derived foodstuffs. *J. Proteomics* **2016**, *147*, 140-155. https://doi.org/10.1016/j.jprot.2016.03.048
[25] Xia, E.-Q; Zhu, S.-S.; He, M.-J.; Luo, F.; Fu, C.-Z.; Zou, T.-B. Marine peptides as potential agents for the management of type 2 Diabetes Mellitus—A prospect. *Mar. Drugs* **2017**, *15*, 88. https://doi.org/10.3390/md15040088
[26] Yan, J.; Zhao, J.; Yang, R.; Zhao, W. Bioactive peptides with antidiabetic properties: a review. *Int. J. Food Sci. Tech*. **2019**, *54(6),* 1909-1919. https://doi.org/10.1111/ijfs.14090
[27] Nauck, M. Incretin therapies: highlighting common features and differences in the modes of action of glucagon-like peptide 1 receptor agonists and dipeptidyl peptase-4 inhibitors. *Diabetes Obes. Metab.* **2016**, *18*, 206-213. https://doi.org/10.1111/dom.12591
[28] McIntosh, C.H.S.; Demuth, H.U.; Pospisilik, A.; Pederson, A. Dipeptidyl peptidase IV inhibitors: how do they work as new antidiabetic agents? *Regul. Pept*. **2005**, *128(2),* 159-165. https://doi.org/10.1016/j.regpep.2004.06.001
[29] Kehinde, B.A.; Sharma, P. Recently isolated antidiabetic hydrolysates and peptides from multiple food sources: a review. *Crit. Rev. Food Sci. Nutr.* **2018**, *21*, 1-19. https://doi.org/10.1080/10408398.2018.1528206
[30] Ramadhan, A.H.; Nawas, T.; Zhang, X.; Pembe, W.M.; Xia, W.; Xu, Y. Purification and identification of a novel antidiabetic peptide from Chinese giant salamander (*Andrias davidianus*) protein hydrolysate against α-amylase and α-glucosidase. *Int. J. Food Prop.* **2017,** *20:sup3*, S3360S3372. https://doi.org/10.1080/10942912.2017.1354885
[31] Lauritano, C.; Ianora, A. Marine organisms with anti-diabetes properties. *Mar. Drugs* **2016**, *14(12),* 220. https://doi.org/10.3390/md141220220
[32] Admassu, H.; Gasmalla, M.A.A.; Yang, R.; Zhao, W. Bioactive peptides derived from seaweed protein and their health benefits: antihypertensive, antioxidant, and antidiabetic properties. *J. Food Sci.* **2018**, *83(1),* 6-16.http://doi.org/10.1111/1750-3841.14011
[33] Giordano, D.; Costantini, M.; Coppola, D.; Lauritano, C.; Núñez Pons, L.; Ruocco, N.; di Prisco, G.; Ianora, A.; Verde, C. Chapter five - Biotechnological applications of bioactive peptides from marine sources. In *Advances in Microbial Physiology*; Poole, R.K., Eds; Publisher: Academic Press, Cambridge, Massachusetts, United States, **2018**, Volume 73, pp. 171-220. http://doi.org/10.1016/bs.ampbs.2018.05.002
[34] Choudhary, A.; Naughton, L.M.; Montánchez, I.; Dobson, A.D.W.; Rai, D.K. Current status and future prospects of marine natural products (MNPs) as antimicrobials. *Mar. Drugs* **2017**, *15(9),* 272. https://doi.org/10.3390/md15090272
[35] Kang, H.K.; Lee, H.H.; Seo, C.H.; Park, Y. Antimicrobial and immunomodulatory properties and applications of marine-derived proteins and peptides. *Mar. Drugs* **2019**, *17*, 350. https://doi.org/10.3390/md17060350
[36] Shannon, E.; Abu-Ghannam, N. Antibacterial derivatives of marine algae: an overview of pharmacological mechanisms and applications. *Mar. Drugs* **2016**, *14*, 81. https://doi.org/10.3390/md14040081

[37] Semreen, M.H.; El-Gamal, M.I.; Abdin, S.; Alkhazraji, H.; Kamal, L.; Hammad., S.; El-Awady, F.; Waleed, D.; Kourbad, L. Recent updates of marine antimicrobial peptides. *Saudi Pharm. J.* **2018**, *26(3*), 396-409. http://doi.org/10.1016/j.jps.2018.01.001

[38] Omardien, S.; Brul, S.; Zaat, S.A. Antimicrobial activity of cationic antimicrobial peptides against gram-positives: current progress made in understanding the mode of action and the response of bacteria. *Front. Cell Dev. Biol*. **2016,** *4*, 111. https://doi.org/10.3389/fcell.2016.00111

[39] Najafian, L.; Babji, A.S. A review of fish-derived antioxidant and antimicrobial peptides: Their production, assessment, and applications. *Peptides* **2012**, *33(1),* 178–185. https://doi.org/10.1016/j.peptides.2011.11.013

[40] Tang, Z.R.; Sun, Z.H.; Tang, X.S.; Feng, Z.M.; Zhou, D.; Xiao, D.F.; Zhang, B.; Li, L.L. Dietary bovine lactoferricin improved gut microflora, benefited intestinal mucosal morphology and inhibited circular cytokines delivery in 21-day weaned piglets. *J. Appl. Anim. Res.* **2011**, *39(2),* 153–157. https://doi.org/10.1080/09712119.2011.565570

[41] Leoni, G.; De Poli, A.; Mardirossian, M.; Gambato, S.; Florian, F.; Venier, P.; Wilson, D.N.; Tossi, A.; Pallavicini, A.; Gerdol, M. Myticalins: A novel multigenic family of linear, cationic antimicrobial peptides from marine mussels (*Mytilus spp.*). *Mar. Drugs* **2017,** *15(8),* 261. https://doi.org/10.3390/md15080261

[42] Kumar, P.; Kizhakkedathu, J.N. ; Straus, S.K. Antimicrobial peptides: diversity, mechanism of action and strategies to improve the activity and biocompatibility *in vivo. Biomolecules* **2018**, *8,* 4. https://doi.org/10.3390/biom8010004

[43] Jiang, Z.; Vasil, A.I.; Hale, J.D.; Hancock, R.E.W.; Vasil, M.L.; Hodges, R.S. Effects of net charge and the number of positively charged residues on the biological activity of amphipathic α-helical cationic antimicrobial peptides. *Biopolymers* **2008**, *90(3),* 369–383. https://doi.org/10.1002/bip.20911

[44] Azoulay, Z.; Rapaport, H. The assembly state and charge of amphiphilic β-sheet peptides affect blood clotting. *J. Mater. Chem. B*, **2016,** *4*, 3859-3867. https://doi.org/10.1039/C6TB00330C

[45] Jung, W.K.; Je, J.Y.; Kim, H.J.; Kim, S.K. A novel anticoagulant protein from *Scapharca broughtonii. J. Biochem. Mol. Biol.* **2002**, *35(2),* 199-205. https://doi.org/10.5483/bmbrep.2002.35.2.199

[46] Jung, W.K., Kim, S. K. Isolation and characterisation of an anticoagulant oligopeptide from blue mussel, *Mytilus edulis. Food Chem.* **2009**, *117*, 687–692. https://doi.org/10.1016/j.foodchem.2009.04.077

[47] Qiao, M.; Tu, M.; Wang, Z.; Mao, F.; Chen, H.; Qin, L.; Du, M. Identification and antithrombotic activity of peptides from Blue Mussel (*Mytilus edulis*) protein. *Int. J. Mol. Sci*. **2018**, *19(1),* 138-149.https://doi.org/10.3390/ijms19010138

[48] Rajapakse, N.; Jung, W.K.; Mendis, E.; Moon, S.H.; Kim, S.K. A novel anticoagulant purified from fish protein hydrolysate inhibits factor XIIa and platelet aggregation. *Life Sci*. **2005**, *76(22),* 2607-2619.https://doi.org/10.1016/j.lfs.2004.12.010

[49] Shimizu, M.; Sawashita, N.; Morimatsu, F.; Ichikawa, J.; Taguchi, Y.; Ijiri, Y.; Yamamoto, J. Antithrombotic papain-hydrolyzed peptides isolated from pork meat. *Thromb. Res.* **2009**, *123(5),* 753–757. https://doi.org/10.1016/j.thromres.2008.07.005

[50] McGraw, A.; Hillarp, A.; Echenagucia, M. Considerations in the laboratory assessment of haemostasis. *Haemophilia* **2010**, *16 (Suppl. 5),* 74–78. https://doi.org/10.1111/j.1365-2516.2010.02302.x

[51] Gils, C.; Vinholt, P.J.; Nybo, M. Falsely prolonged activated partial thromboplastin time – a pre- and post-analytical issue. *Biochem. Med. (Zagreb)* **2019**, *29(1),* 011001. https://doi.org/10.11613/BM.2019.011001
